# Heterogeneities in Axonal Structure and Transporter Distribution Lower Dopamine Reuptake Efficiency

**DOI:** 10.1523/ENEURO.0298-17.2017

**Published:** 2018-02-05

**Authors:** Cihan Kaya, Mary H. Cheng, Ethan R. Block, Tom M. Bartol, Terrence J. Sejnowski, Alexander Sorkin, James R. Faeder, Ivet Bahar

**Affiliations:** 1 Department of Computational and Systems Biology, School of Medicine, University of Pittsburgh, Pittsburgh, PA 15261; 2 Department of Cell Biology, School of Medicine, University of Pittsburgh, Pittsburgh, PA 15261; 3 Department of Science, Chatham University, Pittsburgh, PA 15232; 4 Computational Neurobiology Laboratory, Salk Institute for Biological Studies, La Jolla, CA 92037

**Keywords:** Dopamine reuptake, dopamine transporter, spatial simulation, stochastics

## Abstract

Efficient clearance of dopamine (DA) from the synapse is key to regulating dopaminergic signaling. This role is fulfilled by DA transporters (DATs). Recent advances in the structural characterization of DAT from *Drosophila* (dDAT) and in high-resolution imaging of DA neurons and the distribution of DATs in living cells now permit us to gain a mechanistic understanding of DA reuptake events *in silico*. Using electron microscopy images and immunofluorescence of transgenic knock-in mouse brains that express hemagglutinin-tagged DAT in DA neurons, we reconstructed a realistic environment for MCell simulations of DA reuptake, wherein the identity, population and kinetics of homology-modeled human DAT (hDAT) substates were derived from molecular simulations. The complex morphology of axon terminals near active zones was observed to give rise to large variations in DA reuptake efficiency, and thereby in extracellular DA density. Comparison of the effect of different firing patterns showed that phasic firing would increase the probability of reaching local DA levels sufficiently high to activate low-affinity DA receptors, mainly owing to high DA levels transiently attained during the burst phase. The experimentally observed nonuniform surface distribution of DATs emerged as a major modulator of DA signaling: reuptake was slower, and the peaks/width of transient DA levels were sharper/wider under nonuniform distribution of DATs, compared with uniform. Overall, the study highlights the importance of accurate descriptions of extrasynaptic morphology, DAT distribution, and conformational kinetics for quantitative evaluation of dopaminergic transmission and for providing deeper understanding of the mechanisms that regulate DA transmission.

## Significance Statement

Dopamine (DA) modulates motor control, cognition, and drug addiction. Understanding the mechanism of dopamine transmission is essential to designing therapies for neurologic disorders. We developed a multiscale model using advances in imaging and high-performance computing technologies, which permitted us to perform spatially realistic simulations of dopamine reuptake. Simulations show large temporal and spatial variations in the local density of dopamine depending on the morphology of the synaptic/extrasynaptic region near dopamine release site and on firing pattern. Dopamine clearance is less efficient under heterogeneous distribution of dopamine reuptake transporters, compared with a uniform distribution with the same average surface density. Dopamine reuptake transporters membrane distribution, accessibility of dopamine reuptake transporters outward-facing conformation, and large fluctuations in dopamine levels emerge as key features that modulate dopaminergic transmission.

## Introduction

Midbrain dopaminergic neurons have a strong influence on striatum functions such as motor and action planning, cognitive functions, and motivation (Roeper, 2013). Dysregulation of dopaminergic transmission leads to impairment of these activities, resulting in disorders such as Parkinson’s disease (PD; Hoang, 2014), attention deficit/hyperactivity disorder (ADHD; Wu et al., 2015), and drug addiction (Nutt et al., 2015). A mechanistic understanding of dopamine (DA) transmission events is essential to developing therapeutic strategies, because many behavioral states strongly correlate with DA release and/or reuptake (Tsai et al., 2009; Sulzer et al., 2016).

DA release to the synapse is activated by excitatory stimulation and exhibits patterns similar to neuronal firings. DA excitatory signaling proceeds by activation of DA receptors on binding DA molecules. DA rapidly diffuses from the active zone (AZ; or release site) to extrasynaptic regions in the extracellular (EC) medium. DA transporters (DATs), membrane proteins usually located on the surface of presynaptic axon terminals, regulate DA signaling by removing excess DA from extrasynaptic regions (Torres et al., 2003; Vaughan and Foster, 2013). DATs are targets for addictive substances, which inhibit their function (Amara and Sonders, 1998), thus resulting in excess (neurotoxic) DA levels in the EC region (Di and Imperato, 1988), whereas low levels of DA cause motor impairments associated with PD (Lotharius and Brundin, 2002). In addition, reuptake by DATs, the rate of DA diffusion from the AZ to the extrasynaptic region (Taylor et al., 2013), and the frequency and patterns of action potentials (APs; Tsai et al., 2009) are known to modulate the efficiency of DA signaling.

The dynamics of DA reuptake by DATs has been a focal topic in modeling efforts, at both the cellular and molecular levels (Mortensen and Amara, 2003). Early efforts at the cellular level adopted a well-mixed model focusing on predicting the DA concentration at certain regions of the brain, such as the nucleus accumbens or dorsal striatum (Garris et al., 1994). The effect of EC DA concentration on the activation of DA receptors (Viggiano et al., 2004), as well as spatial/volume exclusion/transmission properties affecting EC DA levels, have been included in later volume transmission (VT) models (Cragg et al., 2001; Cragg and Rice, 2004; Rice and Cragg, 2008; Dreyer et al., 2010; Dreyer and Hounsgaard, 2013; Sulzer et al., 2016). These studies highlighted a need for considering the distinctive diffusion and uptake characteristics of the EC microenvironment. Yet, no quantitative models/simulations have been developed or performed to date that would permit us to assess how the complex geometry of DA terminals and the spatial distribution and conformational dynamics of DATs alter dopaminergic signaling. Advances in imaging DA neurons and visualizing individual DATs (Block et al., 2015) now enable us to reconstruct *in silico* the detailed morphology near AZs and examine the time evolution of DA release and reuptake with the help of MCell, software originally developed (Stiles et al., 1996; Kerr et al., 2008; Czech et al., 2009) for spatiotemporally realistic simulations of synaptic signaling events.

In addition to cellular structure and heterogeneities, the conformational dynamics of DATs is a determinant of DA transport efficiency. Recent crystal structures of *Drosophila* DAT (dDAT; Penmatsa et al., 2013, 2015; Wang et al., 2015) have opened the way to structure-based studies of DAT dynamics. Simulations based on these structures helped elucidate the sequence of molecular events that take place during the transport cycle of the human orthologue, hDAT (Cheng and Bahar, 2015; Khelashvili et al., 2015; Razavi et al., 2017; Cheng et al., 2018). We are now able to make reasonable approximations for the kinetic scheme and parameters associated with the DAT transport cycle based on statistical analyses of the full-atomic trajectories and free energy calculations.

Here, we present an integrated model of synaptic signaling in DA neurons developed from cellular and molecular structures and molecular dynamics. We investigate the effects of (1) the conformational kinetics of DATs, (2) the spatial complexity of DA terminals and AZs based on fluorescence images, (3) the firing patterns, phasic versus tonic, and (4) the heterogeneous distribution of DATs on the plasma membrane based on experimentally observed DAT density fluctuations. Simulations reveal the strong dependence of local DA levels as well as overall DA clearance efficiency on the local geometry of axon terminals. They also reveal that the presence of DAT clusters (consistent with the DAT density heterogeneities observed in high-resolution images; Block et al., 2015) causes a reduction in the efficiency of DA reuptake compared with uniformly distributed DATs with the same average surface density. This effect becomes more pronounced with increasing heterogeneity of the surface distribution of DATs.

## Materials and Methods

### Confocal imaging of immunolabeled DATs in transgenic mouse brains

The procedure for preparing and imaging acute brain slices from transgenic knock-in mice of either sex expressing DAT molecules tagged with the hemagglutinin-A (HA) epitope (HA-DAT; (Rao et al., 2012) has been described in previous works (Rao et al., 2013; Block et al., 2015). Briefly, brains were submerged into an ice slush of oxygenated artificial cerebrospinal fluid (ACSF), and 0.8-mm-thick sagittal slices were cut using microtome blades and a stainless-steel slicing block. The subcellular localization of cell-surface HA-DAT molecules was deduced from intact living DA neurons in acute sagittal brain slices as detected by mouse anti-HA antibodies with Cy3-conjugated anti-mouse antibodies (Block et al., 2015). Slices were incubated in ACSF at room temperature with 1 µg/ml mouse anti-HA antibodies for 1 h. After removing unbound antibodies, slices were incubated for 1 h at 4°C in ACSF with 2.5 µg/ml Cy3-conjugated anti-mouse Fab fragments.

We have not observed substantial differences in HA-DAT distribution between live-cell and postfixation labeling with HA antibodies, suggesting that axonal varicosities revealed by DAT staining were not the result of blebbing during the labeling procedure of live slices. We found that binding of antibodies to live neurons followed by fixation provides much superior image quality and lower signal-to-noise ratio compared with the conventional protocol of fixation first and then staining with antibodies. Importantly, live-neuron staining protocol allows labeling of cell-surface DATs, which is important for defining the distribution of DATs on the neuronal surfaces in the model. Observations of DAT endocytosis (Block et al., 2015), normal lateral membrane mobility of DAT, and healthy mitochondria in dopaminergic neurons from slices kept alive for at least 2 h were indicative of functional neurons in these slices. Moreover, DA neurons labeled as described above have been observed to exhibit pH-dependent vesicular trapping of antipsychotic drugs (Tucker et al., 2015).

To obtain high-resolution 3D images of DA neurons, a *z*-stack of 18 confocal images at 400-nm interstack distance was acquired 10 µm deep from the cut face of the slice through the 561 filter channel using a spinning disk confocal system based on a Zeiss Axio Observer Z1 inverted fluorescence microscope (with 63× Plan Apo PH NA 1.4 objective), equipped with a computer-controlled Spherical Aberration Correction unit, Yokogawa CSU-X1, Vector photo manipulation module, Photometrics Evolve 16-bit EMCCD camera, Hamamatsu CMOS camera, environmental chamber, and piezo stage controller and lasers (405, 445, 488, 515, 561, and 640 nm), all controlled by SlideBook 6 software (Intelligent Imaging Innovation).

Our image reconstruction and modeling described below are based on the combination of light microscopy images of slices and electron microscopy images (Block et al., 2015) that were obtained on intact animals after cardioperfusion fixation.

### *In silico* reconstruction of DA axonal terminals in the striatum

**Figure 1.**
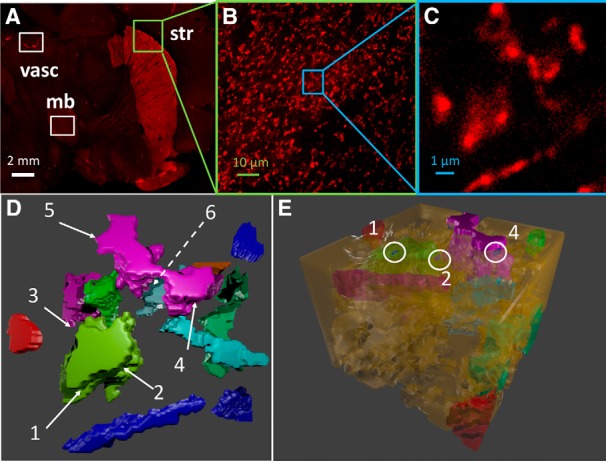
Reconstruction of the morphology of DA neuronal axons. ***A***, HA-DAT distribution in different regions of mouse brain including midbrain (mb) and striatum (str) acquired from sagittal slices. The slices were labeled with HA11 antibody detected with Cy3-conjugated Fab IgG fragments (HA, red). Nonspecific staining of vasculature (vasc) is also highlighted. White scale bar, 2 mm. ***B***, Maximal-projection image of the first five (starting 10 μm deep from the edge of the slice) confocal sections of the 3D image from striatal region. The slice labels are same as those in ***A***. Green scale bar, 10 μm. ***C***, Maximal projection of the first five sections of 3D image of the small striatal region, used to construct the simulation environment (inset), magnified from ***B***. Blue scale bar, 1 μm. ***D***, 3D reconstruction of the region shown in ***C***, visualized using CellBlender (Bartol et al., 2015b), an add-on for Blender 2.78 (http://www.blender.org). Different colors refer to 13 different axonal varicosities (DA terminals). The remaining portions are occupied by cells that do not express DAT. The location of six AZs are shown by the labels 1–6. The dashed line indicates an AZ that is not visible from this perspective. ***E***, Full isometric view of the simulation box. White circles indicate the locations of three AZs.

We reconstructed *in silico* a 10 × 10 × 7.2-μm volume from the above described striatal region (Fig. 1*A–C*) using a semiautomated 3D reconstruction algorithm (Turetken et al., 2016). The size of the simulation box was large enough to allow for diffusion of DA over a sufficiently broad EC region, in accord with previous estimates (Venton et al., 2003), and the reconstruction yielded a realistic representation of both the heterogeneous shape of axonal terminals and the surface distribution of individual DAT molecules. The reconstructed region contained 13 axon terminals (Fig. 1*D*). The corresponding volumes and surface areas (listed in Table 1) were calculated using the NeuroMorph (Jorstad et al., 2015), a Blender add-on that uses triangular meshes to evaluate the surface area and corresponding normal to determine the volume of each tetrahedron. The total volume occupied by the 13 axon terminals was 101.03 μm^3^, and the corresponding total surface area, 337.16 μm^2^.

**Table 1.** Geometric characteristics of axon terminals reconstructed^1^ for simulations

Subsystem	V (μm)^3^	Surface area (μm)^2^	Available^3^ surface area (μm)^2^
DA axons			
Terminal 1	1.22	6.8	5.47
Terminal 2	24.63	59.47	53.83
Terminal 3	3.53	20.18	18.03
Terminal 4	15.84	56.33	51.11
Terminal 5	4.72	25.14	25.14
Terminal 6	1.71	10.12	10.12
Terminal 7	0.82	5.57	5.57
Terminal 8	2.04	12.94	12.94
Terminal 9	15.32	46.86	39.08
Terminal 10	15.18	58.65	55.5
Terminal 11	3.85	18.99	18.98
Terminal 12	9.69	34.31	31.75
Terminal 13	2.58	12.35	9.64
Total for all terminals	101.03	367.71	337.16
Non–DA-expressing cells	463.62	1058.00	0
DA axons + other cells	564.65	1425.71	337.16
Available EC volume^2^	155.35		

1 Using experimental data from Block et al. (2015).

2 Obtained by subtracting 564.65 from 720 μm^3^ (for the simulation box of 10 × 10 × 7.2 μm^3^).

3 Excluding those at the simulation box boundaries.

The DA axonal terminals reconstructed *in silico* contained six varicosities, i.e., 3D globular regions with densely expressed DATs, distributed over three DA terminals: one of the largest terminals had three varicosities, another had two, and the remaining varicosity was on a third terminal. AZs lie within varicosities but are not usually populated with DAT molecules (Block et al., 2015); accordingly, subregions (of varicosities) that lacked DAT molecules within at least a 50-nm radius were identified as AZs. The region between DAT-expressing cells (detected by fluorescence microscopy) and others (not visible) was represented by an interstitial (void) space of 30-nm thickness surrounding the DAT-expressing terminals (Fig. 1*E*). This led to a void fraction of 0.21, consistent with previous estimates (Cragg et al., 2001), or an overall volume of 155.35 μm^3^ (Table 1) that formed available for DA diffusion. These narrow regions form the synaptic clefts and extrasynaptic regions available for the diffusion of DA molecules. The number of AZs for a given volume was verified to be comparable to that used in other studies (Dreyer et al., 2010).

**Figure 2.**
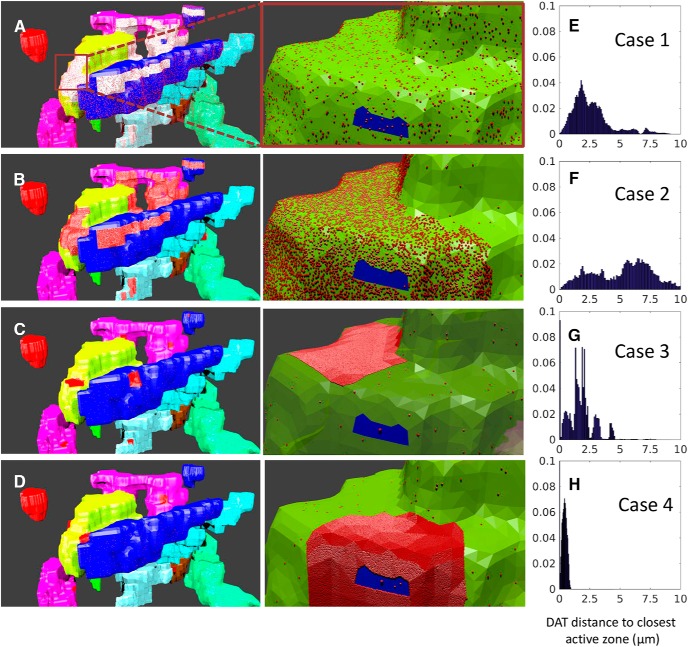
Four space-dependent models of different plasma distributions of DATs. ***A–D***, Distant (left) and magnified (right) view of the axon terminals in cases 1–4. Each color shows a different terminal, and the red dots represent the DATs. The white regions in ***A*** represent the regions with high fluorescence intensity, and those regions are filled with high density of DATs (red dots), magnified in ***B***, where the blue region shows the AZ. The red patches in ***B–D*** illustrate high-density regions where ∼90% of DATs are clustered. ***E–H***, Distance distribution of DATs to closest AZ center, from 140 independent simulations.

Next, we describe the placement of DAT molecules on the membrane of axonal terminals. To investigate the effect of DAT surface distribution heterogeneities on the efficiency of DA reuptake, we examined four cases (Fig. 2). Case 1 refers to the uniform distribution (Fig. 2*A*), taken as ρ(DAT) = 800/μm^2^, based on the electron microscopy images of gold particle labeled HA-DAT, assuming 10% labeling efficiency (Block et al., 2015). Case 2 is a nonuniform (bimodal) distribution (Table 2; Fig. 2*B*), set forth in accord with the actual distribution of DATs observed in experiments. High-density regions were detected in the fluorescence images as continuous bright regions (Fig. 1*C*). These regions covered ∼10% of the plasma membrane area, and ∼90% of DATs were localized in these regions. The surface densities of DATs in the high- and low-density regions were taken as ρ*_h_* = 6339/μm^2^ and ρ*_l_* = 50/μm^2^, respectively. In case 3, the distribution is again bimodal, similar to case 2, but the central parts of the high-density regions from case 2 are selected as the new very-high-density regions, with ρ*_h2_* = 30,000/μm^2^ and ρ*_l_* = 50/μm^2^ elsewhere, which leads to a sharper heterogeneity in the spatial distribution of DATs (Fig. 2*C*). In case 4, DATs are assumed to be clustered in the immediate neighborhood of AZs, as a mimic for conventional synaptic models where DATs act as gatekeepers near the synaptic cleft (Fig. 2*D*; Rothstein et al., 1994; Danbolt, 2001), and DAT surface concentrations in high- and low-density regions are the same as in case 3. The histograms in Fig. 2*E–H* describe the probability distribution of the distances of DAT molecules from the closest AZ.

**Table 2.** Parameters and properties used in MCell simulations

Parameter	Value	Unit
DA diffusion coefficient^a^	4.00 × 10^−6^	cm^2^/s
Vesicle release probability^b^	0.06	
Average firing rate of DA neurons^c^	4.00	Hz
DA released per release event ^d^	3250	*n*
OF → OF* rate constant (*k*_12_)^e^	9.60 × 10^6^	M^−1^s^−1^
OF* → IF* rate constant (*k*_23_)^e^	20.00	s^−1^
IF* → IF rate constant (*k*_34_)^e^	5.00	s^−1^
IF ↔ OF forward rate constant (*k*_41_)^e^	2.00	s^−1^
IF ↔ OF reverse rate constant (*k*_14_)^e^	8.00	s^−1^
Total axonal surface area^f^	337.16	μm^2^
Uniform DAT surface density, ρ(DAT)^f^,^g^	800	1/µm^2^
High DAT surface density, ρ*_*h*_*(DAT)^f^,^h^	6,339	1/µm^2^
Low DAT surface density, ρ*_*l*_*(DAT)^i^	50	1/µm^2^
Very high DAT surface density, ρ*_*h*_*_2_(DAT)^j^	30,000	1/µm^2^
DA density of neurons, I_DA_	9.3	μg/cm^3^
Ratio of the total DA released per AP, R	0.05%	
Density of DA terminals, ρ*_*term*_*	0.104	1/μm^3^

a From Rooney and Wallace (2015).

b Reported in Dreyer et al. (2010)

c See Dreyer et al. (2010).

d See Pothos et al. (1998).

e Estimated from molecular computations.

f Derived from Block et al. (2015).

g Case 1 in Fig. 2.

h High-density region in case 21; see Fig. 2.

i Low-density region in cases 2–4.

j Very-high-density region in cases 3 and 4.

### MCell simulations of DA release and reuptake events in DA neurons

Spatiotemporally realistic simulations were performed using MCell (Stiles et al., 1996; Bartol and Stiles, 2001; Kerr et al., 2008), a 3D reaction-diffusion system solver that allows users to reconstruct complex geometries, define the subcellular localization of discrete molecules, and simulate their dynamics. The parameters used in simulations are given in Table 2. Unimolecular reactions are scheduled according to defined reaction rates, and bimolecular reactions occur with predefined probabilities that are chosen to match bulk reaction rates. Collisions between molecules are detected by ray-tracing algorithm. We adopted Neumann boundary conditions similar to those used in recent simulations of Ca^2+^ signaling (Bartol et al., 2015c), i.e., DA molecules are subjected to reflective boundary conditions at the simulation box walls. In addition, to reduce the bias from reflective boundaries, terminals within 1 μm of the box boundary were assumed to be inactive, such that the available surface area on DA axon terminals was 337.16 μm^2^ (see Table 1).

The probability of a release succeeding an action potential depends on multiple factors (Dreyer et al., 2010), including the content of DA in the striatum (*I_DA_*; Bannon et al., 1981), the ratio of the amount of total DA released per action potential (*R*; Gubernator et al., 2009), the volumetric density of DA terminals at AZs (ρ*_term_*; Doucet et al., 1986), and the number of DA molecules released per quantum (*N*_0_; Pothos et al., 1998). The parameters are given in Table 2, which yielded a release event probability of 6% (Dreyer et al., 2010). Each AZ has a release site located at its center; and on a release event, a total of *N*_0_ DA molecules is assumed to be released from the release site. DA diffusion was modeled as a pseudorandom walk with a fixed time step of Δ*t* = 0.1 µs. The distribution of DA step sizes yielded an average of 13.3 nm using a DA diffusion coefficient of 4 × 10^6^ cm^2^/s. A time step of 100 µs was used for the slow events, such as the transition of DAT to reuptake-ready (EC-exposed outward-facing) state release of its cargo. 140 independent runs, each of 10 s, were performed to extract statistically significant results.

### Conformational dynamics of DAT

**Figure 3.**
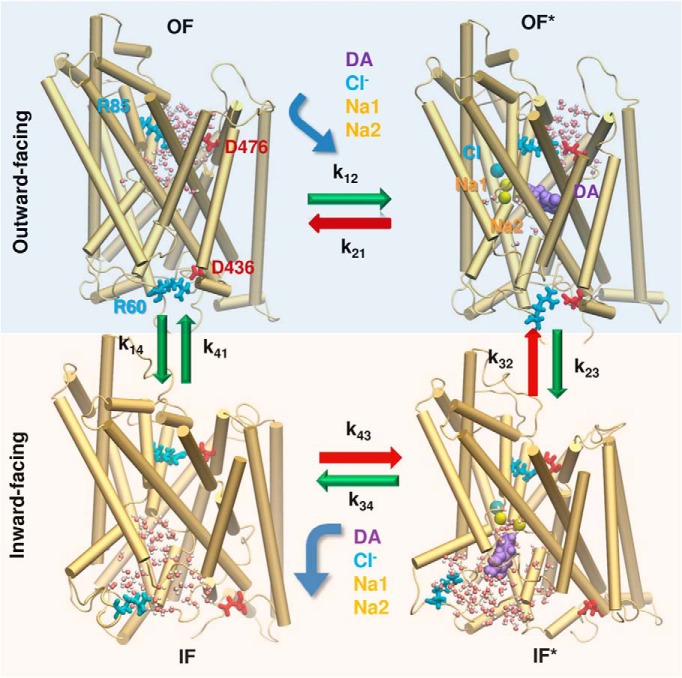
Schematic representation of the sequence of events occurring during the transport cycle of DAT. A succession of four major states is observed in MD simulations: unbound and DA/ion-bound outward-facing states (OF and OF*) followed by unbound and bound inward-facing states (IF and IF*). The corresponding hydration patterns (water molecules in white and pink spheres) and interactions of intra- and extracellular gating residues (R85-D476 and R60-D436, respectively, in stick representation) are displayed. Green arrows indicate the transitions that were observed and evaluated in molecular simulations (see Table 2). The events indicated by the red arrows were unlikely (*k*_21_) or not observed (*k*_32_ and *k*_43_) in MD runs. Curved arrows refer to the binding or unbinding of DA (purple, space filling), cotransported Na^+^ ions Na1 and Na2 (yellow spheres), and the chloride ion (blue sphere).

Our recent dual-boost accelerated molecular dynamics (aMD; Hamelberg et al., 2007; Miao et al., 2013) and conventional MD (cMD) simulations of DAT dynamics showed that the DA transport cycle by DAT can be approximated by four basic steps (Fig. 3; Cheng and Bahar, 2015; Cheng et al., 2018): (1) recognition and binding of DA (and cotransported Na^+^ ions) from the EC region to DAT in the outward-facing (OF) state—we designate the substrate- and Na^+^-bound (or loaded) OF state as OF*; (2) global structural change of DAT from OF* to inward-facing loaded (IF*) state; (3) release of cargo to the IC region (IF* → IF); and (4) reverse transition of the unbound/apo DAT from IF to OF state. The respective forward rate constants are denoted as *k*_12_, *k*_23_, *k*_34_, and *k*_41_, and reverse rate constants are *k*_21_, *k*_32_, *k*_43_, and *k*_14_ (Fig. 3).

The molecular events of DA binding and unbinding to DAT generally involve local conformational changes, and their energetics can be estimated using established free energy calculation methods. We used two methods: (1) alchemical free energy calculation with free energy perturbation (FEP) method (Pohorille et al., 2010) and (2) potential of mean force (PMF) calculations using the adaptive biasing force (ABF) method (Chipot and Hénin, 2005), based on cMD trajectories. The FEP calculations yielded a binding free energy change of Δ*G_bind_* = −7.8 kcal/mol (Cheng et al., 2018), in excellent agreement with the experimental value of −7.4 kcal/mol (Dar et al., 2006; Huang and Zhan, 2007). MD simulations indicated that the average time required to bind a DA molecule originally placed at a distance of 15 Å from the binding site is ∼125 ns. To convert this number into the binding rate constant *k*_12_, we performed the following operation. First, using an EC DA concentration of 7.5 nM (Feifel et al., 2003), we calculated the density of DA molecules to be 7.5 × 10^−9^ mol/nm^3^ × 6.02 × 10^23^ molecules/mol = 4.5 × 10^−9^ molecules/nm^3^. The free volume (excluding that occupied by DAT itself) for DA translocation originally located at a separation of 15 Å from the binding was evaluated to be 2 nm^3^ using POVME (Durrant et al., 2011). The number of DA molecules colliding with DAT based on this accessible volume is 2 nm^3^ × 4.5 × 10^9^ = 9 × 10^−9^, which also represents the *a priori* probability/frequency of collision of a given DA molecule. This leads to an effective binding time of 125 ns/9 × 10^−9^ = 13.88 s. By normalizing with respect to EC DA concentration, the bimolecular reaction constant is determined as (1/13.88 s)/7.5 × 10^−9^ = 9.6 × 10^6^ M^−1^⋅s^−1^.

We further observed that (1) the binding of Na^+^ ions was fast (<100 ns) and the subsequent binding of DA readily prompted the closure of the EC gate such that the escape of DA (and ions) back to the EC region was negligibly small, i.e., *k*_21_ ≪ *k*_12_; (2) no DA efflux to EC region was detected (i.e., *k*_43_ = *k*_32_ ≈ 0); (3) the DA-free (with Na^+^/Cl^−^ bound) OF→IF transition (*k*_14_) was two to three times slower than that in the DA-loaded transition (*k*_23_)—Na^+^- and substrate-binding allosterically promoted a cooperative transition to IF* state (Cheng and Bahar, 2015; Bahar et al., 2015), but such cooperativity was not observed in the apo state; and (4) the DA-free IF→OF transition (*k*_41_) was even slower than the OF→IF transition (*k*_14_) owing to the difficulty in closing the intracellular gates (Cheng et al., 2018). The global OF ↔ IF transition rates were thoroughly examined in microsecond aMD simulations of DA-free DAT, which showed that the population of reuptake-ready (OF) conformers was lower than that of IF (or other intermediate) conformers by a factor or 4, or *k*_14_/*k*_41_ ≈ 4 (Cheng et al., 2018). These considerations provided us with robust information on the relative rates of the individual steps and led to the rate constants in Table 2, the absolute values of which were verified to be compatible with experimentally observed turnover rates and steady-state concentrations of DA molecules.

**Figure 4.**
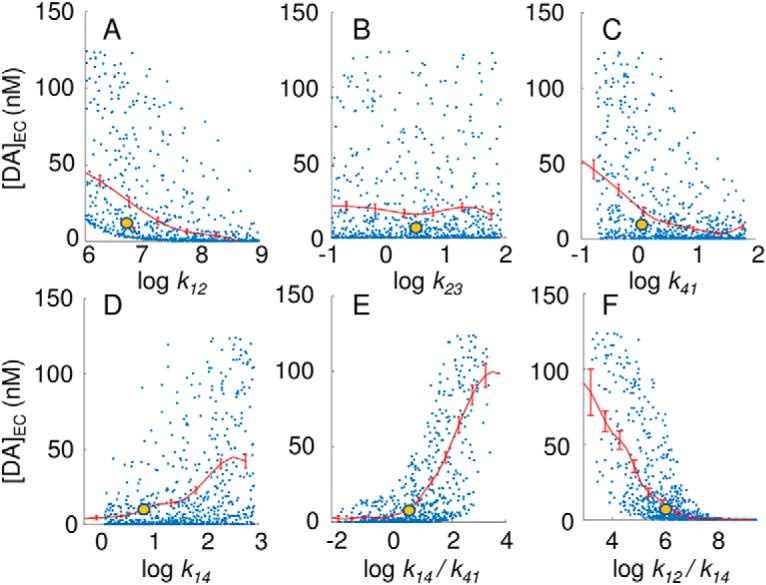
Results from global sensitivity analysis performed for kinetic parameters representing DAT conformational dynamics. Yellow dots represent the default parameters used in the present study, and blue dots show the results obtained by using as input random combinations of the parameters (*k*_12_, *k*_23_, *k*_34_, *k*_41_ and *k*_14_) and their ratios. Results for *k*_34_, which are very similar to those for *k*_23_, are not shown. The red curves indicate the mean values and the standard error for successive bins of width 0.5. The Spearman correlation coefficients are –0.71, 0.01, –0.01, –0.44, 0.44, 0.63, and –0.48, in the six respective panels.

To investigate the sensitivity of DA reuptake efficiency to DAT conformational kinetics, we also performed global sensitivity analysis with respect to rate constants in Fig. 3. We performed 729 independent runs with different combinations of *k*_12_, *k*_23_, *k*_34_, *k*_41_, and *k*_14_, which we varied by three orders of magnitude. The results are presented in Fig. 4. Each blue dot represents the outcome, EC DA concentration in the simulation box, [DA]_EC_, from one run. A broad range of [DA]_EC_ values, from 0.1 to >100 nM, are observed, yet an increase in DA binding rate *k*_12_ results in a more efficient clearance and thereby lower DA levels in the EC region (Fig. 4*A*). A similar trend is detected with an increase in the transition rate *k*_41_ from IF to OF, which exposes more reuptake-ready DATs to the EC region (Fig. 4*C*), and the reverse transition induces the opposite effect (Fig. 4*D*). An even sharper effect is observed on plotting [DA]_EC_ against the ratio *k*_14_/*k*_41_, highlighting the importance of the equilibrium population of the OF and IF states of DAT after releasing its cargo (Fig. 4*E*). The examination of the relative effects of DA binding (*k*_12_) versus back-transition to the IF state (*k*_14_) for the OF DAT also indicates that the OF DAT level is a major determinant of [DA]_EC_ (Fig. 4*F*)_._ Further quantitative assessment of the statistical significance of these observations using Spearman rank correlation coefficients confirmed that the binding rate constant *k*_12_ and the ratio *k*_14_/*k*_41_ are two major determinants of DA clearance efficiency. No clear effect was seen for *k*_23_ (Fig. 4*B*) or *k*_34_ (data not shown).

### Code accessibility

The MCell software and the input files for the spatial models implemented in MCell are freely available to the community. The model files developed in this study can be accessed as Extended Data 1 or GitHub repository at https://github.com/cihankayacihan/dopamine_striatum_mcell. The only requirement for repeating the simulations is to download the MCell software (http://www.mcell.org) version 3.4 and use it together with the model files. The available files include information on geometry, molecules, reactions, surface classes, and release patterns in addition to output trajectories and MCell simulation parameters.

10.1523/ENEURO.0298-17.2017.1 Extended Data 1 Code: MCell models are described by mdl files. Mdl files are modular and information related to geometry, molecules, reactions, surface types, release events, and output are given in the code. Input files could be modified using any text editor. The main file needs to be executed with MCell. Directories with names react_data and viz_data are created that contain the simulation output described in the input files. Download Extended Data 1, ZIP file.

## Results

### *In silico* turnover and [DA]_EC_ at half-maximal rate conform to experimental data

We first verified that MCell simulations yielded macroscopic properties consistent with experimental data. We calculated the turnover rate by adopting in our simulations the same protocol as that adopted in experiments (Rao et al., 2013): multiple runs are performed for a series of initial concentration of DA in the EC region, [DA]_0_, and in each case, the mass of DA molecules transported per unit time is measured. The number of DA molecules transported per second, V_max_, under saturation conditions ([DA]_sat_ is of the order of tens of micromoles per liter) is used to evaluate the turnover rate as the ratio of V_max_ to the total number B_max_ of DAT molecules present in the system. In our simulation environment, B_max_ ≈ 220,000, based on fluorescence microscopy data (Block et al., 2015). To evaluate the turnover rate *in silico*, we counted the number of DAs transported as a function of [DA]_0_ and examined for each concentration the number of DAs translocated per second. This led to a reuptake rate of 1.2 × 10^5^ DAs/s at saturation (V_max_). Division by B_max_ gave a turnover rate of 0.55/s, which is comparable to the reported values of 0.2/s (Rao et al., 2013), 0.9/s (Prasad and Amara, 2001), and 1.8/s (Prasad and Amara, 2001; Beuming et al., 2008; Rao et al., 2013).

**Figure 5.**
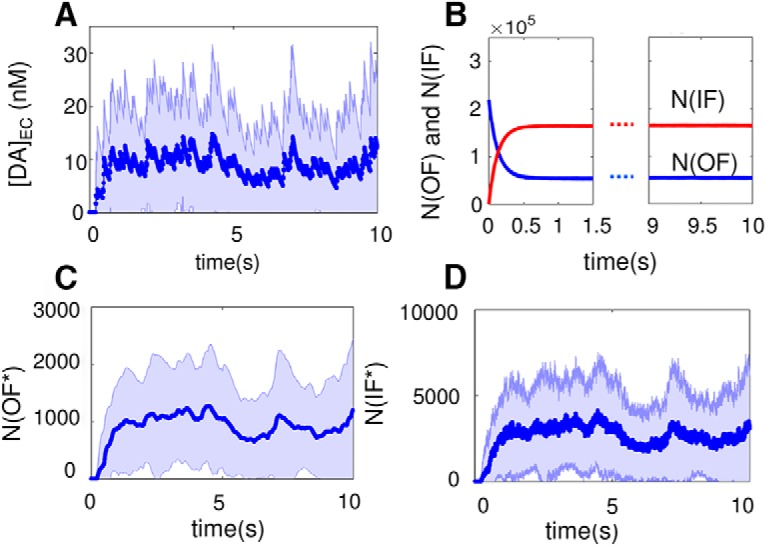
Time evolution of DA concentration and DAT conformational states averaged over 140 independent MCell runs. ***A***, Extracellular DA concentration, [DA]_EC_. The average concentration reached under steady-state conditions is 7.8 nM, and the standard deviation of the concentration is indicated by the shaded region is Δ[DA]_EC_ ≈ 10 nM. ***B***, The number of DAT molecules, in the unbound inward- or outward-facing state (IF: red; OF; blue), denoted as N(IF) and N(OF), respectively, as a function of time. ***C***, ***D***, The average numbers of DATs in substrate-bound OF* and IF* states, N(OF*) and N(IF*), respectively. The light blue bands show the variance observed in multiple simulations.

The average DA level in the EC medium, [DA]_EC_, observed *in silico* after reaching steady-state conditions was 7.8 nm (Fig. 5*A*). The physiologic concentration of DA in the striatum varies between 5 and 50 nm (Owesson-White et al., 2012), consistent with the large fluctuations (of the order of Δ[DA]_EC_ ≈ ±10 nm) we observed in [DA]_EC_. Note that the saturating concentration for DATs is estimated to be ∼10 μm (Prasad and Amara, 2001; Rao et al., 2013). Simulations yielded a substrate concentration at half-maximal rate, *K_m_* value, of 2.2 μm, which falls within the broad range of reported experimental values of 50 nM to 6.6 μm (Prasad and Amara, 2001; Beuming et al., 2008; Rao et al., 2013). These data confirm that MCell model and simulations reproduce macroscopic quantities consistent with observables such as the average DA concentration in the EC region at half-maximal rate and the overall turnover rate. Next, we make a closer examination of microscopic properties.

### DAT conformers reach a dynamic equilibrium within 100s of milliseconds

**Figure 6.**
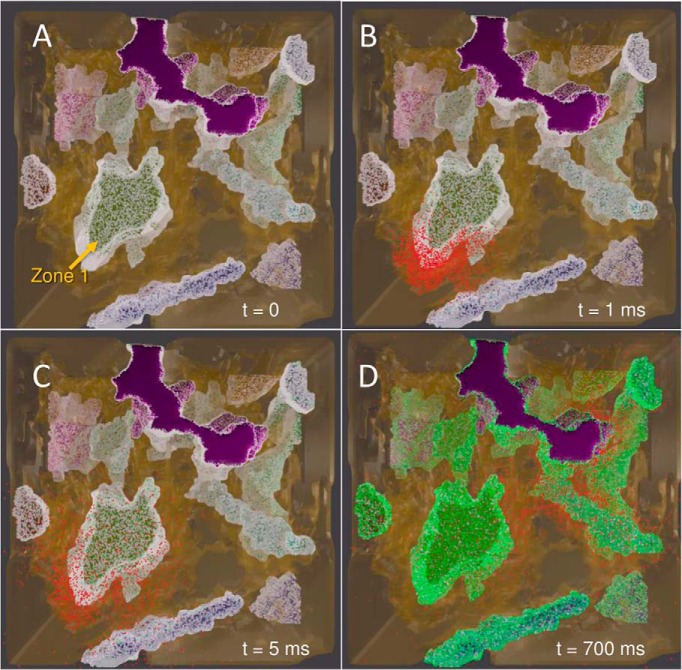
Snapshots from MCell simulations of DA release and reuptake by DATs on DA terminals. Snapshots from an equilibration simulation of 1 s, initiated by a release event at *t* = 0, and followed by AP firings at 4-Hz frequency are displayed, visualized using Blender. Color code: red, DA; white, OF DAT; green, IF DAT. The purple region shows an axon terminal that is inactive during the simulations. Initially, all DATs are in the OF state (***A***). A release event at 1 ms is shown in ***B***, and another at 5 ms (***C***), where most of the DATs reside in the OF state. DA molecules diffuse to extrasynaptic regions in <10 ms. ***D***, The high population of DATs in the IF state reached at ∼700 ms.

First, we examined the equilibration of the simulated system under uniform surface distribution of DAT molecules on the axonal membrane. The four snapshots in Fig. 6 (and Video 1) illustrate the initial DA release events and the gradual equilibration of the conformational states of DATs. All DAT molecules are assumed to be in the OF state at *t* = 0 (white dots on the surface of the terminals). Simulations start with a first release event (at AZ 1; Fig. 6*A*), followed by firings with Poisson distribution. The released DA molecules (red dots) rapidly diffuse to the vicinity of the release site, as illustrated in the snapshots at *t* = 1 and 5 ms (Fig. 6*B*,*C*). At *t* = 700 ms, we observe a broad spatial distribution of DA (Fig. 6*D***)**.

Video 1. DA release, diffusion, and reuptake events in the presence at the early (equilibration; 100s of milliseconds) and later (up to 4 s) of MCell simulations. Color code: red, dopamine; green, inward-facing DAT; white, outward-facing DAT. For the first 10 ms, interframe time interval is 1 ms; between 10 and 1000 ms, it is 10 ms; in the rest, 100 ms. To emphasize release events, the frequency of snapshots is reduced between 3.5 and 4 s. Multiple release events are observed, including one at 3.8 s where a closeup view of the corresponding AZ is displayed. 10.1523/ENEURO.0298-17.2017.video.1

Fig. 6*D* shows that most of DATs reside in the IF state (colored green) at *t* = 700 ms. This is consistent with the equilibrium probabilities of the four DAT conformers (19.86% OF, 79.90% IF, 0.05% OF*, and 0.19% IF*), which is reached within 500 ms. Fig. 5*B–D* displays the time evolution of the population of the different states of DAT, averaged over 140 independent runs of 10-s duration each. Most of the DATs fluctuate between unbound OF and IF states, whereas the bound states (OF* and IF*) are short-lived. Because of their scarcity, the numbers of DATs in IF* and OF* states show significant fluctuations during simulations (indicated by the light blue band in Fig. 5*C*,*D*).

### DA levels exhibit large fluctuations depending on AZ structure and DAT surface distribution

**Figure 7.**
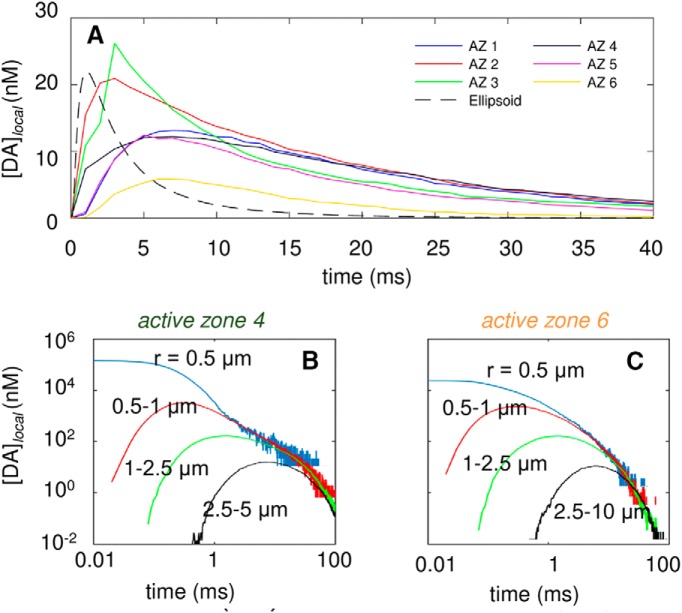
Time evolution of DA levels in the EC region after a single release event in different AZs. ***A***, Time evolution of EC DA levels within a spherical shell of inner radius *r_i_* = 1 μm and outer radius *r_o_* = 2.5 μm centered at the AZ or top of the ellipsoid for six different AZs and ellipsoid geometry with same volume and surface area (labeled AZ1–AZ6 and ellipsoid, shown in different colors and patterns; see inset labels). ***B***,***C***, Local concentration of DA within spherical regions of radius 0.5 μm (blue) and within spherical shells of 0.5 ≤ *r* < 1 μm (red), 1 ≤ *r* < 2.5 μm (green), and 2.5 ≤ *r* < 5 μm (black), near AZ4 (***B***) and AZ6 (***C***), as labeled.

We present in Fig. 5*A* the time course of the DA concentration in the EC region, [DA]_EC_, averaged over 140 runs, which exhibited a standard deviation of ∼10 nm about the mean value of 7.8 nm. These are global fluctuations, i.e., they refer to the average behavior of the overall microenvironment simulated by MCell. A look at local time- and space-resolved patterns of DA levels, on the other hand, reveals even broader fluctuations depending on the AZ and the particular space or time window examined. The curves in Fig. 7*A* display the time evolution of the local concentration, [DA]*_local_*, within a spherical shell of 1.5-μm thickness, with respective inner and outer radii of 1 and 2.5 μm (i.e., 1 ≤ *r* < 2.5 μm) centered at the release site, after a release event from the release site on each of the 6 AZs. Results represent the averages over multiple release events (140 per AZ) observed within the time interval 0 < *t* < 40 ms.

A considerable variation in the maximum level of DA reached and in the succeeding decay rate is observed in Fig. 7*A*, depending on the AZ that discharges the DA molecules. For AZ 2 and AZ 3, [DA]*_local_* can reach 20–25 nm, but for AZ 6 it remains <7 nm. AZ 2 and 3 are on a surface-exposed region of axon terminal 2, in close proximity of non–DAT-expressing cells (Fig. 1*C*). AZ 6, however, lies inside a cavity on axon terminal 4 that harbors a large density of DATs (Fig. 1*C*), hence the rapid depletion of the released DA. Other AZs (1, 4, and 5) show an intermediate behavior, because although they are exposed to the EC region, the DAT level in their vicinity is also relatively high (according to fluorescence images).

To examine the effect of neuronal region structural complexity, computations were repeated for a hypothetical EC region with a simplified (ellipsoidal) geometry that preserved the same EC volume and neuronal surface area as those of the simulated system, together with the same number of DAT molecules on the surface, thus maintaining the same DAT surface density as the above system. DA reuptake evaluated for the same spherical shell (Fig. 7*A*, dashed black curve) centered around the release site was observed to be faster than that occurring in the realistic environment. The faster removal of DA molecules is due to the more efficient diffusion of DA in this hypothetical environment devoid of irregularities/obstacles.

To make a further quantitative assessment of the role of the AZ geometry and local structural heterogeneity in affecting the different time evolutions of DA clearance observed in Fig. 7*A*, we examined the tortuosity and void fraction in the vicinity (within 2.5-μm radius) of each of the 6 AZs. The results are presented in Table 3. Both properties affect [DA]*_local_*: the clearance of DA is less efficient when the void fraction is low, and the tortuosity is high (e.g., AZ 3 followed by AZ 2). Although void fraction appears to have a dominant effect, tortuosity becomes an important factor when distinguishing the rise time and peak height between AZs that exhibit similar void fractions. AZ 6 has a high void fraction and a low tortuosity, which leads to a rapid reuptake of DA. Another variable we examined is the distance to the closest DAT (Table 3), but it does not have a substantial effect because the diffusion is rapid.

**Table 3.** Structural properties near AZs^a^

Active zone	Void fraction	Tortuosity	Distance to closest DAT (μm)
1	0.35	1.22	0.20
2	0.22	1.38	0.21
3	0.20	1.56	0.24
4	0.31	1.28	0.20
5	0.37	1.36	0.89
6	0.36	1.23	1.22

a Within a spherical region of 2.5-μm radius centered around the release site.

Overall, these results show that (1) the different time evolution of [DA]*_local_* in response to DA release from different AZs can be traced back to differences in tortuosity and void fractions and (2) the adoption of uniform tortuosity and void fraction for all AZs would not provide a realistic representation of the heterogeneous synaptic structure, nor would it account for the differential reuptake efficiency originating from these local geometric effects.

Fig. 7*B*,*C* further illustrates the variations in [DA]*_local_* within gradually increasing distance ranges with respect to the release sites in AZs 4 and 6. [DA]*_local_* within a spherical volume of radius *r* = 0.5 μm remains >10 μm for ∼1 ms after DA release from AZ 4, and that within 0.5 ≤ *r* < 1.0 μm rises more slowly and temporarily reaches 1 μm (Fig. 7*B*). Comparison with AZ 6 shows the lower [DA]*_local_* near the release site (*r* < 0.5 μm) and the depletion of DA at t ≈ 100 ms (Fig. 7*C*). The large variations in [DA]*_local_* due to stochasticity and heterogeneity of the release sites occur during the entire course of the simulations.

The variations in [DA]*_local_* are critically important because, although [DA]_EC_ may not be sufficient for activating DA receptors, [DA]*_local_* or the corresponding fluctuations Δ[DA] may bring the local DA concentration above the threshold levels required for activating even low-affinity receptors. These results underscore the significance of considering the space-dependent, stochastic nature of DA density fluctuations for estimating the probability of DA receptor activation in complex microenvironments.

### Cellular structural complexity modulates the fractional occupancy of high-affinity DA receptors

Dopaminergic signals are transmitted on DA receptor activation. There are five different types of DA receptors, commonly classified as D1-like (D1, D5), which have low-affinity for DA (EC_50_ ∼1 μm), and D2-like (D2, D3, D4), which have high affinity (EC_50_ ∼10 nm; Rice and Cragg, 2008). As shown in Fig. 7*B*,*C*, [DA]*_local_* may reach 10–100 μm near an AZ, immediately after vesicular discharge, and low-affinity receptors may be activated if they are in proximity. However, DA molecules rapidly diffuse from the release site and the synaptic cleft (of ∼0.25–0.5-μm radius) to extrasynaptic regions, such that synaptic [DA] decreases by ∼3 orders of magnitude within tens of milliseconds.

**Figure 8.**
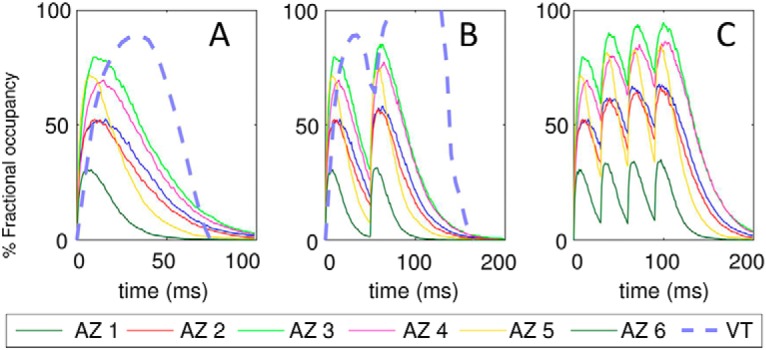
Expected fractional occupancy (or percentage saturation) of DA high-affinity receptors in response to successive releases. Fractional occupancy as a function of time is based on the probabilistic occurrence of saturation conditions ([DA]_local_ ≥ 10 nM) in the synaptic region associated with each AZ. Results are shown for different AZs (as labeled) in response to single release (***A***), two releases with 50-ms interspike interval (***B***), and four releases with 30-ms interspike intervals (***C***). Results from the VT model are shown for comparison in ***A*** and ***B***.

We evaluated the fractional occupancy *f*(*t*) of high-affinity DA receptors after a release event for each of the 6 AZs. For the calculation of occupancy, high- and low-affinity receptors were assumed to be uniformly distributed on the membrane of all pre- and postsynaptic cells, similar to previous approaches (Dreyer et al., 2010). Note that the activation of low-affinity receptors requires high [DA]*_local_* values, and such high concentration levels are temporarily attained only in the proximity of the release sites. So, in practice, only those low-affinity receptors located sufficiently close to the AZs will be activated. As for high-affinity receptors, they are likely to be activated at all locations. To evaluate the fractional occupancy of high-affinity DA receptors for each of the 6 AZs, we analyzed 140 × 6 = 840 runs as follows: we divided the simulation box into cubic grids of 1 fl (10^−15^ L), and we counted the number of DA molecules in each cube, to obtain the local concentrations. If the latter was zero at all time points, the cube was labeled as an excluded volume (inaccessible to DA molecules); otherwise, the probability of satisfying the threshold level (10 nM) for high-affinity receptors was evaluated for each AZ as a function of time elapsed after DA release. The results are presented in Fig. 8*A*. *f*(*t*) temporarily reaches a maximum *f_max_* before it decays to near zero within ∼100 ms. *f_max_* shows a strong dependence on the release site, varying from 0.27 (site 6) to 0.78 (site 3), again indicating the importance of the local environment.

To compare our results with those predicted by the VT model, we first evaluated the two parameters of the model: the tortuosity of the terminals and the void fraction of cytoplasmic regions accessible to DA diffusion (Dreyer et al., 2010). To this aim, we randomly selected 5000 pairs of mesh points and evaluated the ratio of their shortest arc distance along the surface to the corresponding Euclidean distance. This gave an average tortuosity of 1.79. The void fraction was 0.21, as noted above (Table 1). The results are displayed by the dashed curves in Fig. 8*A*,*B*.

Fig. 8*A* reveals several differences between the predictions of the VT model and those obtained by MCell simulations. The VT model predicts a higher percentage saturation (based on 10-nm threshold value), a slower binding kinetics (indicated by the longer time to reach the maximal occupancy), and faster decay kinetics, compared with MCell predictions. Furthermore, the VT model reaches saturation conditions after two releases with 50-ms spike interval (Fig. 8*B*)—a level not reached in MCell simulations even after four spikes with interspike interval shortened to 30 ms (Fig. 8*C*). Finally, the VT model cannot capture the variations in [DA]*_local_* depending on the AZs (or their specific structure or nearby DAT density).

### Phasic firing favors high transient levels in [DA] while retaining the average [DA]_EC_

**Figure 9.**
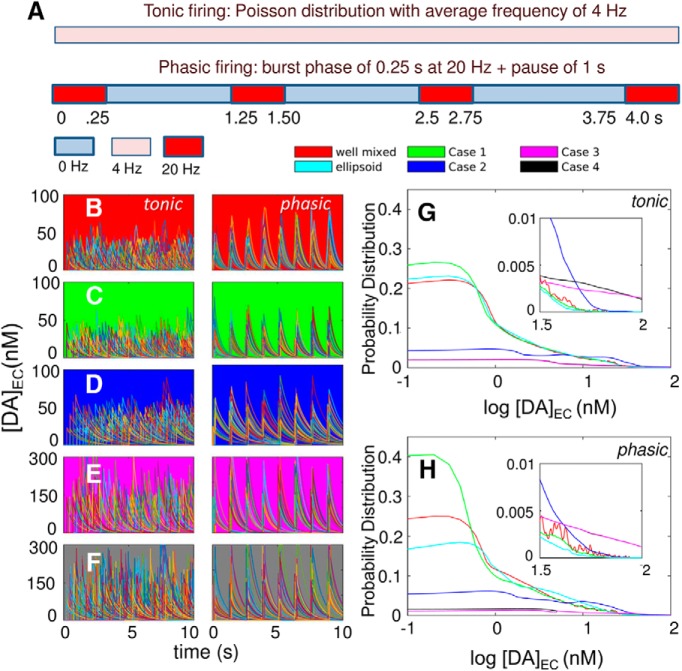
Comparative analysis of global EC DA levels under different firing patterns and the effect of the complexity of cell geometry and the heterogeneity of DAT surface density. ***A***, Schematic description of the firing patterns: tonic (upper bar) and phasic (lower bar). Each color represents a different firing frequency. Tonic firing has a constant vesicular release frequency of 4 Hz throughout the complete duration of the simulations (here shown for 4 s). Phasic firing has burst periods of 0.25 s with a firing frequency of 20 Hz separated by pauses of 1 s. ***B–F***, Time evolution of [DA]_EC_ for five models: well-mixed (background colored red); MCell with uniform distribution of DATs (background in green); MCell with nonuniform distributions of DATs (cases 2–4 in Fig. 2; background in blue, magenta, and gray, respectively), presented for tonic (left) and phasic (right) firing patterns. Each panel displays the results from 140 individual trajectories from independent runs. ***G***,***H***, Probability distributions of log [DA]_EC_ level observed in different runs, shown for tonic (***G***) and phasic (***H***) firing patterns. The respective insets show the behavior in the range log[DA_EC_/nM] > 1.5. Results for ellipsoid geometry are presented in cyan. The principal semi-axis lengths of the ellipsoid are 8.73, 1.44, and 4.36 μm, which yield the same surface area and volume with the realistic geometry.

We investigated the effect of distinctive release patterns, tonic and phasic (Goto et al., 2007), on DA reuptake efficiency. Tonic firing was implemented as random spikes generated from a Poisson distribution at 4-Hz frequency (Dreyer et al., 2010), and phasic firing consisted of a burst phase for 0.25 s at 20-Hz frequency followed by a pause of 1 s (Fig. 9*A*), both yielding the same average frequency. The time evolution of [DA]_EC_ was examined under those two firing patterns for five different models: a well-mixed system (Fig. 9*B*) and four spatially realistic models derived from image data (cases 1–4 in Fig. 2), which differ in the membrane distribution of DATs. The cases are (1) uniform (Fig. 9*C*), (2) nonuniform (Fig. 9*D*), and (3 and 4) sharply nonuniform with two different localizations of high-density regions (Fig. 9*E*,*F*; see Table 2 for corresponding surface densities). Each panel displays 140 curves, each corresponding to an independent run.

Tonic and phasic firing patterns elicit markedly different fluctuations in [DA]_EC_. Tonic firing shows irregular fluctuations (left panels in Fig. 9*B–F* and Video 2), which usually remain <60 nm in Fig. 9*B–D*, whereas phasic firing in the same cases (right panels and Video 3) easily exceeds 60 nm, especially when the surface density of DATs is nonuniform (Fig. 9*D*). Sharply nonuniform distributions of DATs (Fig. 9*E*,*F*) give rise to increased [DA]_EC_, while the difference in the fluctuation behavior of [DA]_EC_ under tonic and phasic firing persists. These results suggest that phasic firing could more readily favor low-affinity DA receptor activation, although the average [DA]_EC_ values over the entire duration of simulations are comparable (Table 4), consistent with the same average firing frequency shared between the five cases.

Video 2. Dynamics of DA molecules and time evolution of [DA]_EC_ under tonic firing. Same color code as in Video 1. The total duration of the movie is 5 s. The bottom plot displays the time evolution of [DA]_EC_ during the course of simulations. 10.1523/ENEURO.0298-17.2017.video.2

Video 3. Dynamics of DA molecules and time evolution of [DA]_EC_ under phasic firing. Same color code as in Video 1. The time span of the movie is 5 s. 10.1523/ENEURO.0298-17.2017.video.3

**Table 4.** [DA]__EC__values (mean ± SEM) under different conditions

Space	DAT distribution	[DA]_EC_ ± Δ [DA]_EC_ (nM)	
		Tonic	Phasic
Well-mixed DA^a^		8.16 ± 0.92	8.29 ± 1.41
Ellipsoid geometry		8.21 ± 0.85	8.33 ± 1.44
Complex space			
Case 1	Uniform^b^	7.54 ± 0.86	6.93 ± 1.92
Case 2	Nonuniform^c^	16.59 ± 1.26	14.19 ± 2.44
Case 3	Sharp bimodal^d^	33.12 ± 3.13	29.65 ± 4.49
Case 4	Sharp bimodal^d^	32.49 ± 2.86	32.89 ± 3.68

a Space/structure-independent. All other cases are in realistic DA neuronal geometry.

b DAT densities for uniform and nonuniform (bimodal) distributions; see Table 2.

c Case 2 (ρ*_h_* = 6339/μm^2^ and ρ*_l_* = 50/μm^2^).

d Cases 3 and 4 (ρ*_h_* = 30,000/μm^2^ and ρ*_l_* = 50/μm^2^; see Figs. 2 and 11).

**Figure 10.**
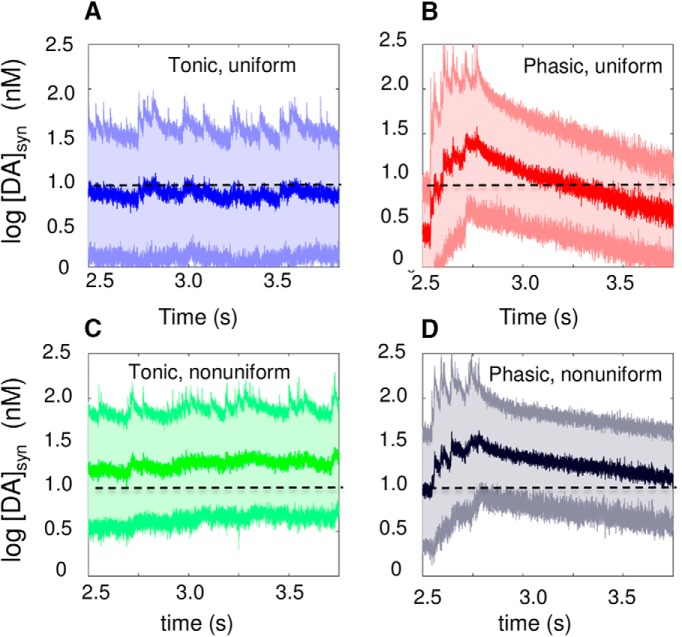
Time evolution of DA level in the synapse after a release event. ***A–D***, [DA]_syn_ as a function of time for tonic (left) and phasic (right) firing, under uniform (top) and nonuniform (bottom) distribution of DATs. The mean and variation in [DA]_syn_ within 1 μm collected from 140 independent runs are displayed. Results are shown for the time interval 2.5–3.75 s as a single firing block, representative of dynamic equilibrium reached under stationary conditions. The thick line in the middle of cloud is the mean value over all runs, and the cloud represents the standard deviation, highlighting the large fluctuations in the synaptic DA levels.

A closer examination of the transient DA levels within the synaptic region reveals the difference between phasic and tonic firing. We defined [DA]_syn_ as the transient DA level within a sphere of 0.5-μm radius centered at a release site and examined [DA]_syn_ after a single release event. Fig. 10 displays the mean (central dark curve) and the standard deviation (light curves and shade) in [DA]_syn_ for tonic (Fig. 10*A*,*C*) and phasic (Fig. 10*B*,*D*) firing, under uniform (case 1; Fig. 10*A*,*B*) and nonuniform (case 2; Fig. 10*C*,*D*) distributions of DATs. Similar to the results for the overall EC (synaptic and extrasynaptic) region (Fig. 9*C*,*D*), phasic firing temporarily leads to a higher accumulation of DA in the synapse compared to tonic, which can more readily activate the high-affinity DA receptors (the dashed line indicates the threshold concentration, 10 nm, for their activation).

Finally, we computed the fractional occupancy of low-affinity receptors (Vivo et al., 2006; Casadó et al., 2009) during the burst and pause periods of phasic firing and overall period of tonic firing. The results summarized in Table 5 show that the probability of reaching the threshold DA level of 1 μm for binding low-affinity DA receptors is enhanced by a factor of 2–5 in the burst phase of phasic firing, compared with tonic firing. Further computations by adopting a Michaelis–Menten type occupancy model with half-maximal substrate concentration (*K_m_*) of 10 nm for high-affinity receptors and 1 μm for low-affinity receptors confirmed the same behavior. In the latter model, the occupancy of DA receptors as a function of [DA]*_local_* is given by

$$\theta \left({\left[\mathrm{DA}\right]}_{\mathrm{local}}\right)=\frac{{\left[\mathrm{DA}\right]}_{\mathrm{local}}}{K_{m}+{\left[\mathrm{DA}\right]}_{\mathrm{local}}},$$

and the results in the last column of the Table 5 are obtained on integration over snapshots at 1-μs intervals in 140 trajectories.

**Table 5.** Percentage of high- and low-affinity DA receptors that can potentially bind DAs^a^

			Percent probability of DA binding to DA receptors	
DAT distribution	DA receptor type	Firing pattern and stage	Based on saturation threshold levels	Based on Michaelis–Menten kinetics
Uniform (case 1)	High affinity			
		Tonic	40.26	45.98
		Phasic burst	45.09	51.80
		Phasic pause	4.54	50.07
	Low affinity			
		Tonic	0.40	2.22
		Phasic burst	1.10	4.64
		Phasic pause	0.06	1.92
Nonuniform (case 2)	High affinity			
		Tonic	71.14	63.84
		Phasic burst	65.88	62.70
		Phasic pause	72.05	64.5
	Low affinity			
		Tonic	0.35	3.47
		Phasic burst	1.97	5.47
		Phasic pause	0.03	2.97

a The burst period is 2.5–2.75 s after DA release, and the pause 2.75–3.75 s (see Fig. 10).

### Nonuniform surface distribution of DATs is a major modulator of the strength and intensity of DA signaling

Results in Fig. 9 and Table 4 reveal the strong dependence of DA clearance efficiency on DAT surface distribution. DA reuptake is significantly more efficient with a uniform surface distribution of DATs than with a nonuniform distribution, as evidenced by the relative heights of the peaks at low concentrations in the histograms displayed in Fig. 9*G*,*H* and the average [DA]_EC_ values listed in Table 4. We investigated the differences among well-mixed, simple spatial (ellipsoid), and complex realistic geometries while keeping the EC volume, surface area, and total density of DAT fixed. Results suggest that the approximation of the axonal morphology by a simplified (ellipsoidal) geometry yields results comparable to those predicted by a well-mixed model. Inclusion of realistic geometry, on the other hand, leads to a broad range of results depending on the specific AZ (Figs. 7 and 8) and the surface distribution of DATs (Fig. 9*G*,*H*).

The most efficient clearance is observed in MCell simulations with uniform distribution of DAT. The bimodal distribution of DATs, on the other hand, shows a distinctive distribution of [DA]_EC_ skewed toward higher concentrations, or a significantly suppressed DA reuptake efficiency, as can be seen more clearly in the insets, irrespective of the firing pattern (Fig. 9*G*,*H*). This effect becomes more pronounced with increasing heterogeneity of DAT distribution, i.e., going from case 2 to cases 3 and 4.

**Figure 11.**
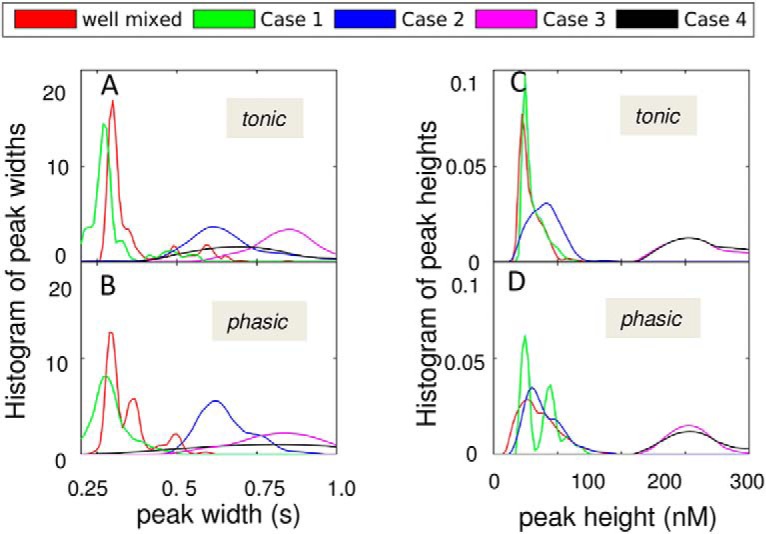
Distribution of peak heights and widths for EC DA levels. Results are presented for four different types of DAT distribution on the plasma membrane, in addition to the well-mixed case. The curves are color-coded, as labeled. The probability distributions of peak widths (***A*** and ***B***) and peak heights (***C*** and ***D***) are presented for tonic (tonic) and phasic (bottom) firing patterns.

For a more critical assessment of the duration and intensity of excitatory stimulation induced in response to the two firing patterns, and under different spatial distributions of DATs, we analyzed the peak heights and widths in Fig. 9*B–F*. The results in Fig. 11 confirm that DAT spatial distribution is a major determinant of dopaminergic signaling strength and duration, whereas the effect of different firing patterns is moderate. In particular, the histograms of the peak widths in Fig. 11*A*,*B*, and peak heights in Fig. 11*C*,*D* clearly show that bimodal DAT distribution (black and magenta curves) leads to more sustained and stronger excitations compared to uniform (or well-mixed) cases (green and blue curves), originating from less efficient removal of DA. Note that the bimodal distribution was selected to mimic the physiologic density heterogeneities observed in high-resolution images (Fig. 1 and Table 2). The effect of DAT density heterogeneity, manifested by an overall suppression in DA reuptake, is further accentuated in sharply heterogeneous distributions of DATs (cases 3 and 4). The most probable peak heights in those cases are of the order of hundreds of nanomoles per liter and may result in neurotoxicity. Taken together, these results demonstrate that heterogeneity of DAT density can modulate DA signaling.

A comparison between cases 3 and 4 further shows that the placement of DATs in the close neighborhood of the AZs does not increase clearance. The diffusion of DA is fast enough to sample extrasynaptic regions, and the localization of DATs with respect to the AZ has a minimal effect. These results suggest that the mechanism of local clearance as envisioned by the gatekeeper model (Rothstein et al., 1994) does not necessarily increase reuptake efficiency.

**Figure 12.**
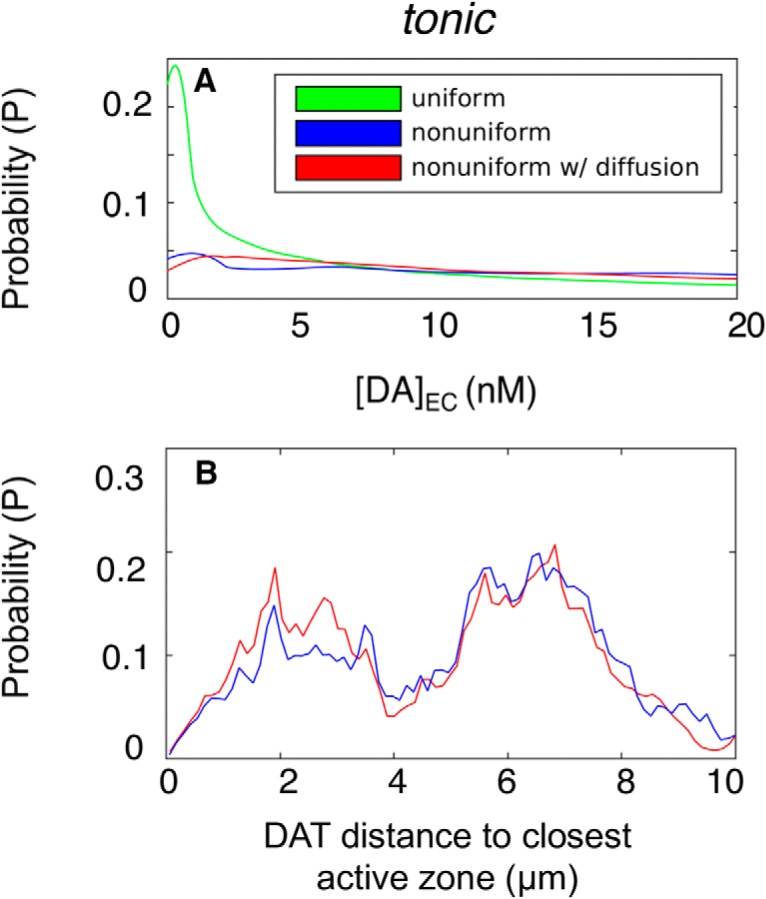
***A***, Probability distributions of [DA]_EC_ level under lateral DAT diffusion, shown for tonic and [DA]_EC_. Green, blue, and red represent uniform DAT distribution, nonuniform DAT distribution, and nonuniform DAT distribution with lateral diffusion of DAT, respectively. ***B***, Probability distributions of DAT distances to closest AZ from 140 independent simulations for the nonuniform DAT distribution with (red) and without (blue) lateral diffusion.

To examine the change in DA reuptake behavior possibly induced by DAT 2D displacement, we repeated the simulations by allowing DAT 2D diffusion with a coefficient of 3 × 10^−10^ cm^2^/s on the plasma membrane (Fig. 12). Comparison of the initial and final (at the end of 10-s simulations) averaged over 140 independent runs showed that no significant change occurred in the position of DATs, owing to the slow diffusion of DATs compared with the time scale of simulations. Overall, these results show that increasing heterogeneity in the surface distribution of DAT leads to lower DA clearance efficiency and/or stronger and more sustained signaling.

## Discussion

### Overview

In the present study, we reconstructed *in silico* the 3D structure of DA terminals, the location of AZs, and the spatial distribution of DATs based on electron and fluorescence microscopy data with transgenic mice (Block et al., 2015) and conducted a series of MCell simulations under different conditions. We determined the spatiotemporal distribution of DA molecules in the synapse and extrasynaptic regions in response to different firing patterns and in the presence of different surface distributions of DATs. These simulations show the effects of the synaptic and extrasynaptic morphology, as well as the heterogeneity of DAT surface distribution (as observed in experiments), on the overall efficiency of DA clearance and the probability of activating high- and low-affinity receptors. Heterogeneous distribution of DATs is shown to reduce the clearance efficiency, the effect being sharper with increased heterogeneity, and not affected by DAT 2D diffusion on the plasma membrane. Although DA dynamics is diffusion-controlled in general, a global sensitivity analysis indicated that the DA-binding rate of DAT and the relative population of its outward- and inward-facing conformers in the apo state (denoted as OF and IF) had major effects on DA reuptake efficiency. Overall, the results underscore the utility of conducting spatiotemporally realistic simulations in elucidating the dependence of dopaminergic signaling on both the surface distribution and conformational mechanics of DATs.

Recent MCell simulations of glutamatergic signaling based on EM images of hippocampal neuropil for investigation of the effect of spatial heterogeneities (Kinney et al., 2013; Bartol et al., 2015a, c) also indicated the importance of including the microenvironment for an accurate description of the electrophysiology and biochemistry of neurotransmission events. No such simulations had been performed for DA neurons to date, mainly because of the lack of high-resolution image data of DA terminals for reconstituting *in silico* the simulation environment. Recent advances in imaging DA neurons (Block et al., 2015) permitted us to overcome this barrier. Furthermore, improved understanding of the structural dynamics of DAT (Cheng and Bahar, 2015; Cheng et al., 2015; Gur et al., 2017) helped us build a simplified kinetic scheme (Fig. 3) that we adopted in MCell simulations.

### DA reuptake simulations require spatially extended (∼10^3^-μm^3^) structural models

The simulation of dopaminergic signaling events presents new challenges compared with that of neurotransmission in glutamatergic synapses. The transporter cycle rate in the latter case is ∼35 glutamate molecules/s per glutamate transporter, whereas the rate in dopaminergic neurons is 1–5 DA molecules/s per DAT (Wadiche et al., 1995; Povlock and Schenk, 1997; Prasad and Amara, 2001; Rice et al., 2011). Thus, simulation of DA clearance requires longer computing times. In addition, glutamate transporters are localized in pre- and postsynaptic regions and mostly in the surrounding glia in the close neighborhood of the AZ (Seal and Amara, 1999; Danbolt, 2001). DATs, on the other hand, are preferentially located in the extrasynaptic regions of the axons and dendrites, and most of the reuptake events take place at sites distal from the AZs (Nirenberg et al., 1996; Rice et al., 2011; Block et al., 2015). Realistic simulations of such spatially distributed events require the adoption of model systems composed of multiple synapses, multiple AZs, and heterogeneous distributions of multiple DAT clusters. We reconstructed *in silico* a relatively large (10 × 10 × 7.2-µm) striatal region.

### Spatial irregularities and hindrance in the interstitial region between neurons limits DA receptor activation

Our simulations show that under conditions that reproduce physiologic levels of DA and turnover rates (Prasad and Amara, 2001; Beuming et al., 2008; Rao et al., 2013), the complex geometry of DA terminals modulates the occupancy of high-affinity DA receptors to <100%, even under sustained, elevated stimulation conditions (Fig. 8*A–*C), consistent with the concept of a dead space (Kamali-Zare and Nicholson, 2013). In contrast, less detailed partial differential equation models (Dreyer et al., 2010) and algebraic models (Cragg and Rice, 2004) predict full occupancy of high-affinity receptors under the same conditions. This difference invites attention to the effect of EC spatial irregularities on dopaminergic signaling efficiency in the brain (Syková and Nicholson, 2008).

### Heterogeneous surface distribution of DAT reduces the effectiveness of DA clearance

EM and fluorescence images showed that the distribution of DAT is heterogeneous in different parts of the striatum (Block et al., 2015). Recent studies indicate that the population of DATs may vary depending on the membrane curvature (Caltagarone et al., 2015). Notably, selected DAT mutants that have disrupted OF conformations do not accumulate in filopodia, suggesting that access to the OF/OF^*^ state may be a prerequisite for DAT to populate the filopodia (Caltagarone et al., 2015). Further examination showed that binding of cocaine and its fluorescent analog JHC1-64 also alters the plasma membrane distribution of both wild-type and mutant DATs. Cocaine binding arrests DAT in the OF state, and cocaine-bound DATs predominantly localize in the filopodia (Ma et al., 2017). Likewise, zinc binding, also known to stabilize the OF state, led to an increase in the level of DAT mutants in the filopodia. These observations suggest that the membrane curvature is a determinant of DAT clustering, with the convex shape of the filopodia favoring the localization of OF DATs. Furthermore, clustering of OF DATs could remodel the membrane to induce an overall outward bending (Ma et al., 2017). The present study suggests that such targeting of axonal protrusions by OF DATs, along with their membrane-remodeling capacities, may regulate DA reuptake.

The total number of DAT molecules was sufficiently large in current simulations (∼200,000) to ensure normal DA clearance consistent with physiologic levels. However, simulations repeated with the same number of DATs distributed nonuniformly on the neuronal membranes showed a reduction in reuptake capacity that became more pronounced with increasing surface density heterogeneity. The surface distribution of DATs indeed emerged as a major determinant of the efficiency of reuptake, or conversely, the strength and duration of excitatory signaling (Figs. 9 and 11).

In principle, one might expect that the increased population of DATs in dense regions would counterbalance the effect of lower surface area coverage on DA reuptake efficiency. However, DA diffuses fast enough to escape from these dense regions after a few encounters, resulting in reduced DA reuptake. These observations further support the importance of adopting a realistic representation of the heterogeneity of DAT surface distribution, especially with low copy numbers of neurotransmitters, for a realistic assessment of DA reuptake capacity.

### Firing patterns determine the relative levels of inhibitory versus excitatory responses

Our simulations revealed that the average [DA]_EC_ exhibited little dependence on firing pattern, provided that the average firing frequency was maintained, but the local levels within a synapse or close to DA release sites exhibited strong dependencies. The size of the fluctuations, Δ[DA], in DA levels were relatively small in tonic firing but large in phasic firing, especially at the burst phase and under sustained phasic firing (Fig. 9*B–F*). Low-affinity DA receptors need two orders of magnitude larger DA concentrations than high-affinity receptors to get activated. As a result, the activation of low-affinity receptors is limited, if not unlikely, with tonic firing (Table 5). Phasic firing, on the contrary, induces a larger variance in both global and local DA concentrations and is two to five times more likely to activate low-affinity receptors during the burst phase of DA signaling (Table 5). Previous work showed that the D1 receptors are mostly localized at the postsynaptic membrane (Cadet et al., 2010), and our simulations show that only those low-affinity receptors localized near the synapse can be activated. In the case of high-affinity receptors, on the other hand, proximity to release site is not a requirement for being activated: high-affinity receptors located on distal regions may be equally activated.

DA receptors regulate the activity of DA neurons. One distinction between high- and low-affinity DA receptors is the type of response they elicit in the cell, inhibitory or excitatory (Keeler et al., 2014), after their activation on DA binding. D1-like receptors, which are usually low-affinity receptors, are involved in excitatory signaling, whereas D2-like receptors trigger inhibitory signaling processes. High-affinity receptors may be activated by both phasic and tonic firing. However, our simulations suggest that low-affinity receptors would be rather activated on phasic firing.

Among D2-like receptors, D2 autoreceptors are known to be key regulators of DA transmission, located on most axon terminals (Ford, 2014). Although D2-like receptors are usually high-affinity receptors, D2 autoreceptors exist in both high- and low-affinity states, and recent studies indicate that the functionally relevant D2-autoreceptors are predominantly in low-affinity state. Their inhibitory action is particularly important to suppressing DA release from presynaptic cells under prolonged bursts of action potentials. They suppress DA release in the striatum by several mechanisms, e.g., by inhibiting the voltage-gated calcium channels that trigger exocytotic DA release or increasing DAT activity or surface expression (Ford, 2014). Previous studies suggested that tonic firing does not raise [DA]_EC_ to levels sufficiently high to activate D2-autoreceptors (Beckstead et al., 2007; Ford, 2014). The low probability of binding low-affinity receptors under tonic firing demonstrated in the present simulations is consistent with the functioning of D2-autoreceptors as low-affinity receptors.

A previous model modified the probability of DA release on an action potential according to high-affinity receptor occupancy (Dreyer and Hounsgaard, 2013). In our current model, receptor occupancy has not been coupled to DA release and reuptake. However, our modeling framework allows for implementing a mechanistic model that incorporates the mediation of DA release and DAT expression by D2-autoreceptors. Such an extension may be key to improved interpretation of DA reuptake dynamics at longer time scales and under disease states.

### The modeling framework is extensible to analyzing the effect of psychostimulants on DA reuptake dynamics

DAT kinetics is often described by the alternating access model (Vaughan and Foster, 2013). Most cell-level models of DA transport at the cellular level assume an immediate transition from IF to OF state right after DA release to the cell interior (Cragg and Rice, 2004; Rice and Cragg, 2008; Sulzer et al., 2016), and the transition from OF to IF in the unbound form is not explicitly considered. In contrast, molecular simulations revealed a complex dynamics (Cheng and Bahar, 2015), which we reduced to a four-state kinetic model (OF, IF, OF*, and IF*). The translocation and release events after substrate binding are much faster compared with the return of apo IF state to OF state and succeeding substrate binding event, which depends on prior probabilities of encounters between DAT and DA molecules. These features result in short lifetimes for the bound forms of DAT and the dominance of the relative populations of the unbound OF and IF states in determining the overall clearance rate (Fig. 5). In particular, the balance between the OF and IF states (or the ratio of the associated rate constants, *k*_14_/*k*_41_, using detailed balance principle) emerged as a major determinant of reuptake efficiency, as it directly defines the fraction of reuptake-ready (OF) DATs.

Previous x-ray crystallographic studies (Penmatsa et al., 2015; Wang et al., 2015) as well as structure-based simulations (Cheng et al., 2015) have shown how the conformational state of DAT and substrate binding affinity may change in the presence of different antidepressant and psychostimulants drugs, such as cocaine and amphetamine (AMPH). Antidepressants usually arrest DAT in OF state [and the same effect has been observed for the homolog serotonin transporter in the presence of antidepressants (S)-citalopram or paroxetine (Coleman et al., 2016)], thus preventing the progress of the transport cycle, and causing an increase in EC DA levels. On the other hand, the occupancy of the Zn^2+^-binding site (Stockner et al., 2013; by zinc or other transition metals) reduces the DA binding affinity of DAT (Li et al., 2017). The current modeling framework can readily be extended to incorporate such effects through suitable readjustment of kinetic parameters in Fig. 3.

AMPH, on the other hand, appears to be a substrate for DAT that competes with DA for transport (Zhu and Reith, 2008). AMPH is also known to promote both DA efflux (reverse transport from the cell interior to the EC region) and DAT internalization (Wheeler et al., 2015). The current simulations focused on the influx of DA from the EC region to the presynaptic cell interior, but the modeling framework is readily extensible to simulating DA efflux as well. Other extensions of the present framework could incorporate the effects of DAT internalization, membrane polarization (Richardson et al., 2016), or perturbations at the membrane-proximal N-terminal residues (Sorkina et al., 2009). Thus, the present study opens the way to quantitative modeling of the effects of antidepressants, psychostimulants, and substances of abuse on the deregulation of DA signaling.

## Synthesis

Reviewing Editor: Miriam Goodman, mbgoodman@stanford.edu Decisions are customarily a result of the Reviewing Editor and the peer reviewers coming together and discussing their recommendations until a consensus is reached. When revisions are invited, a fact-based synthesis statement explaining their decision and outlining what is needed to prepare a revision will be listed below.

I want to thank you for your patience during the review process. One factor that delayed this decision was that the two reviewers who assessed your study had divergent opinions about the significance and the advances relative to a prior study. After soliciting additional input from the reviewers, I'm pleased to offer you the opportunity to provide a revision. In your revision, please focus your efforts on directly addressing the influence of morphology on the dynamics of DA signaling (Reviewer #1, point 4) or refrain from making claims that this study yields new insight on this question. I also request that you address the minor comments from both reviewers regarding the presentation of the work, paying close attention to improving readability regarding abbreviations for model parameters.

Reviewer #1

The authors used MCell to create an in silico model of realistic dopamine terminal geometries obtained from their own experiments; further, this group previously reduced the complexity of DAT conformational changes to 4 states. They modeled the spatiotemporal dynamics of dopamine concentration in the extracellular space following tonic or phasic firing of dopamine neurons.

While they present an important step forward in modeling of extracellular dopamine concentration, I do not think this paper is suitable for publication in a general neuroscience journal like eNeuro.

Major concerns:

1. The authors intended to take important steps in aligning the modeling of dopamine concentration with physiological parameters - they use realistic morphology of dopamine terminals and the main contribution of this work is considering the spatiotemporal dynamics of dopamine diffusion on transporter and receptor occupancy. However, the quality of the images was not high. Indeed the representative image show axonal 'blebbing.'

2. On the experimental methodology, it is not clear why the authors chose to image 10 µm from the cut surface when their sections are 800 µm thick; the terminals near the cut surface may be damaged. They treated fresh slices with antibodies at room temperature, and then 4 C in ACSF with Cy3-conjugated antibody. Then, the slices were used for confocal imaging. This seems very unusual. Why not using fixed tissue? The quality of the brain slices quickly declines over a few hours. It is hard to imagine that one could obtain high quality confocal images from brain slices that have been treated for at least 2 hours with antibodies.

3. The main contribution of this work has already been published by Cragg et al. in 2001. The addition of the DAT kinetic model does not add biological insight.

4. Also, the authors underutilized the power of their sophisticated model - the authors claim that the specific geometry of the axonal terminals play a major role in the dynamics of the dopamine concentration. However, they never explicitly tested this in their model - they could have kept the total surface area or volume constant but altered the geometry of the terminals with the same DAT distributions to make this claim.

5. Further, the authors did not use the model to answer the "why" question: why would dopamine terminals have a heterogenous DAT distribution when the model reveals this distribution has a lower efficiency of uptake efficiency.

Finally, many acronyms and terms not commonly encountered by a general neuroscience audience are lacking and there are many grammatical errors and typos in the text.

Reviewer #2

The manuscript describes modeling the spatiotemporal dynamics of the concentration of dopamine released from axon terminals as a function of action potential firing pattern, distance from the release site, the distribution of DAT, and the transport cycle of dopamine through DAT conformations. The data are discussed in the framework of dopamine D1 and D2 receptor activation. The simulations will contribute to the understanding of dopamine levels, especially as more is known about the location of release sites and dopamine receptors.

The following points should be clarified in the text:

1.When initially introduced, [DA]EC, [DA]local, and [DA]syn should be delineated in terms of distance from the release site and time from release event, in the main text.

2.The “active zones” described in the manuscript are not unequivocal release sites, but assumed to be dopamine release sites. Without the inclusion of more data to this end, this point should be made clear in the text. In addition, the sentence on page 6 “AZs lie within varicosities but are not usually populated with DAT...” should have a reference.

3.It is unclear where postsynaptic D1 and D2 receptors are being assumed to be localized relative to the release site. In the absence of data demonstrating their location (e.g. in brain slices), these assumptions should be described clearly and referenced, in the Results and Discussion.

Minor points:

1.The introduction “low levels of DA cause PD” should be changed to “low levels of DA cause motor impairments associated with PD”.

2.It is unclear to what “DA density of the neurons” on page 7 is referring.

3.Page 15, “Table 5” should read “Table 3”

4.Discussion of D2 autoreceptor activation/occupancy would broaden the scope and readership of this manuscript.

## Author Response

Response to Reviewers

In the following, the Reviewers' comments are written in black font, our responses in blue, and the excerpts from the revised manuscript are indented and written in blue, smaller font.

Reviewer #1:

The authors used MCell to create an in silico model of realistic dopamine terminal geometries obtained from their own experiments; further, this group previously reduced the complexity of DAT conformational changes to 4 states. They modeled the spatiotemporal dynamics of dopamine concentration in the extracellular space following tonic or phasic firing of dopamine neurons.

While they present an important step forward in modeling of extracellular dopamine concentration, I do not think this paper is suitable for publication in a general neuroscience journal like eNeuro.

Response: We thank the reviewer for carefully reading our manuscript and acknowledging that our work represents 'an important step forward in modeling of extracellular dopamine concentration'. We have performed additional computations, and presented additional results along the comments suggested by Reviewer 1, so as to make our work accessible and interesting to a broad neuroscience audience.

Major concerns:

1. The authors intended to take important steps in aligning the modeling of dopamine concentration with physiological parameters - they use realistic morphology of dopamine terminals and the main contribution of this work is considering the spatiotemporal dynamics of dopamine diffusion on transporter and receptor occupancy. However, the quality of the images was not high. Indeed the representative image show axonal 'blebbing.'

Response: We thank the reviewer for pointing out the poor quality of images shown in our submission and the potential for experimental artifacts. It should be emphasized that our images are of diffraction-limited resolution and of high signal-to-noise ratio, which could be achieved only using our imaging protocol (see below). In the revised Figure 1B, the scale bar is shown, so that it is clear to the reader that that the resolution is ~ 250 nm. The single-confocal section image is replaced by the maximal projection image of the first five sections of the 3D image, which offers a better and more representative way of visualizing DAT localization. Further, our image reconstruction and modeling are based on the combination of light microscopy images of slices and electron microscopy images (Block et al., 2015) that were obtained on intact animals after cardioperfusion fixation. We have not observed substantial differences in HA-DAT distribution between live-cell and post-fixation labeling with HA antibodies, suggesting that axonal varicosities revealed by DAT staining are not the result of blebbing during the labeling procedure of live slices. In fact, we can readily see blebbing in the first layer of cells from the cut (within first 10 µm from the cut surface of the z-stack of images), which is morphologically dissimilar from the axonal varicosities in the intact striatum (beyond first 10 µm from the cut).

These points are incorporated in the manuscript on page 5:

We have not observed substantial differences in HA-DAT distribution between live-cell and post-fixation labeling with HA antibodies, suggesting that axonal varicosities revealed by DAT staining were not the result of blebbing during the labeling procedure of live slices. We found that binding of antibodies to live neurons followed by fixation provides much superior image quality and lower signal-to-noise ratio as compared to the conventional protocol of fixation first and then staining with antibodies. Importantly, live-neuron staining protocol allows labeling of cell-surface DATs, which is important for defining the distribution of DATs on the neuronal surfaces in the model. Observations of DAT endocytosis (Block et al., 2015), normal lateral membrane mobility of DAT, and healthy mitochondria in dopaminergic neurons from slices kept alive for at least two hours were indicative of functional neurons in these slices. Moreover, DA neurons labeled as described above have been observed to exhibit pH-dependent vesicular trapping of antipsychotic drugs (Tucker et al., 2015).

And page 6:

Our image reconstruction and modeling described below are based on the combination of light microscopy images of slices and electron microscopy images (Block et al., 2015) that were obtained on intact animals after cardioperfusion fixation.

2. On the experimental methodology, it is not clear why the authors chose to image 10 Âµm from the cut surface when their sections are 800 Âµm thick; the terminals near the cut surface may be damaged. They treated fresh slices with antibodies at room temperature, and then 4 C in ACSF with Cy3-conjugated antibody. Then, the slices were used for confocal imaging. This seems very unusual. Why not using fixed tissue? The quality of the brain slices quickly declines over a few hours. It is hard to imagine that one could obtain high quality confocal images from brain slices that have been treated for at least 2 hours with antibodies.

Response:

1) We indeed find that the first layer of cells from the cut is damaged (see previous comments), and therefore we “discard” the first 10 µm from the cut surface of the z-stack of images (see Materials and methods, last paragraph on page 5, beginning of page 6). Thus, for modeling we used a representative region of the 3D image that begins from 10 µm from the slice surface and is 7.2 µm deep.

2) In previous work (Block et al., J. Neurosci., 2015) and unpublished experiments, it is found that binding of antibodies to live neurons followed by fixation provides much superior image quality and lower signal-to-noise ratio as compared to the conventional protocol of fixation first and then staining with antibodies. The latter procedure involves fixation with para-formaldehyde that by itself partially permeabilizes membranes, affects membrane and general cell morphology, results in significant non-specific binding of antibodies, and reduces the penetration of antibodies into the tissue because of fixation of extracellular matrix. Importantly, live-neuron staining protocol allows labeling of cell-surface DATs which is important for defining neuronal surfaces in the model.

3) We certainly agree that slices deteriorate with time, and the electrophysiological measurements are highly sensitive to this process. We maintain slice oxygenation and osmolarity (see Block et al.), and observed DAT endocytosis, lateral membrane mobility and healthy mitochondria in dopaminergic neurons from slices kept alive for at least 2 hours. Dopamine neurons labeled in this manner have been observed to exhibit pH-dependent vesicular trapping of antipsychotic drugs (Tucker, Block et al., 2015 PNAS).

We have included in the text a summary of the points described above (see response to item 1), together with the revised Figure 1 (and caption) and the reference to previous work (Tucker et al, Proc Natl Acad Sci USA 2015) cited above.

3. The main contribution of this work has already been published by Cragg et al. in 2001. The addition of the DAT kinetic model does not add biological insight.

Response: We respectfully disagree on both points.

First, the model introduced by Cragg et al. reduces the geometric complexity to two parameters - tortuosity and void fraction. Our computations performed in response to this comment clearly show that the different active zones or the vicinity of release sites exhibit significant differences in their tortuosity and void fraction and the adoption of the same parameters for all release sites would not provide an accurate description of the heterogeneity of the environment. We have now created a new table, Table 4, where we have listed the tortuosity and void fraction values for each of the active zones that we have reconstructed in silico, using image data from experiments. The table clearly shows the broad variation in those two properties, and the inadequacy of adopting unified tortuosity and void fraction for all regions of the DA neurons in the striatum. In fact, the distribution of curves in Figures 7 and 8 clearly shows how the DA release from different sites exhibit different kinetics.

Second, Cragg et al. also do not consider the effect of heterogeneity in the membrane distribution of DATs, which we found to be a major factor affecting the rate of reuptake (Figures 9, 11 and 12). One of the major results from the current study is indeed the decrease in reuptake efficiency upon increase in the heterogeneity of the surface distribution of DAT.

Third, as we showed in Fig. 5, the DA reuptake is differentially sensitive to the conformational kinetics of DAT. The model identifies the DA binding rate to outward-facing DAT and the equilibrium constant between unbound inward- and outward-facing DAT to be important determinants of reuptake dynamics. Simple kinetic models do not take account of the availability of outward-facing or inward-facing conformers of DAT in simulating DA binding.

4. Also, the authors underutilized the power of their sophisticated model - the authors claim that the specific geometry of the axonal terminals play a major role in the dynamics of the dopamine concentration. However, they never explicitly tested this in their model - they could have kept the total surface area or volume constant but altered the geometry of the terminals with the same DAT distributions to make this claim.

Response: We appreciate this comment and we have performed additional computations to demonstrate the power of the model.

Before proceeding to the new results, we would like to point out that the data presented in Figures 7 and 8 already showed how the specific geometry of the axonal terminals near the release sites affected the dynamics of dopamine concentration (Figure 7) and the probability of binding high-affinity receptors in response to successive releases (Figure 8). Distinctive curves were observed for the time evolution of DA levels and receptor occupancy for each of the release sites. Given that these differences originated from differences in both specific geometry and the surface distribution of DATs near the different release sites, we performed additional computations, to clarify the effect of specific geometry, alone. To this aim we performed two sets of computations (A) and (B), described below:

(A) As described in the reply to Comment #3 above, we have analyzed the geometry around each active zone and calculated the tortuosity, void fraction and closest distance to a dopamine transporter. We have added a table showing these parameters (Table 3) and added extra text about the effect at the end of page 12. We found that the specific geometry near the different active zones cannot be represented by two parameters, tortuosity and void fraction, shared by all active zones. On the contrary, the parameters that best represent the different active zones vary widely between the active zones. These results are described in the newly added text copied below (from page 14)

To make a further quantitative assessment of the role of the AZ geometry and local structural heterogeneity in affecting the different time evolutions of DA clearance observed in Figure 7A, we examined the tortuosity and void fraction in the vicinity (within 2.5 µm radius) of each of the 6 AZs. The results are presented in Table 3. Both properties affect [DA]local: the clearance of DA is less efficient when the void fraction is low, and the tortuosity is high (e.g. AZ 3, followed by AZ 2). While void fraction appears to have a dominant effect, tortuosity becomes an important factor when distinguishing the rise time and peak height between AZs that exhibit similar void fractions. AZ 6 has a high void fraction and a low tortuosity which leads to a rapid reuptake of DA. Another variable we examined is the distance to the closest DAT (Table 3), but it does not have a substantial effect since the diffusion is rapid.

Overall these results show that (i) the different time evolution of [DA]local in response to DA release from different AZs can be traced back to differences in tortuosity and void fractions, and (ii) the adoption of uniform tortuosity and void fraction for all AZs would not provide a realistic representation of the heterogeneous synaptic structure, nor would it account for the differential reuptake efficiency originating from these local geometric effects.

(B) We used an ellipsoid geometry to represent the extracellular volume; we placed DATs uniformly on that surface; and we have maintained the total extracellular volume, the density of transporters and the total surface area of the original simulation environment, as suggested by the Reviewer, to allow for direct comparison of the results from this hypothetical simplified geometry and those obtained using the realistic geometry. The time evolution of local DA concentration, [DA]local, obtained for the ellipsoidal geometry (newly added dashed black curve in Figure 7A) significantly differs from those obtained for the different release sites in our MCell geometry, as summarized in the text copied below (end of page 13)

To examine the effect of neuronal region structural complexity, computations were repeated for a hypothetical EC region with a simplified (ellipsoidal) geometry that preserved the same EC volume and neuronal surface area as those of the simulated system, together with the same number of DAT molecules on the surface, thus maintaining the same DAT surface density as the above system. DA reuptake evaluated for the same spherical shell (Figure 7A, dashed black curve) centered around the release site was observed to be faster compared to that occurring in the realistic environment. The faster removal of DA molecules is due to the more efficient diffusion of DA in this hypothetical environment devoid of irregularities/obstacles.

We have further examined the DA clearance efficiency observed in MCell simulations and that obtained with the simplified geometry. The new results are shown by the new curves (colored cyan) added to panels G and H of Figure 9, so as to facilitate the comparison. Ellipsoidal geometry gives results similar to those obtained for non-spatial (well-mixed) systems (red curves); whereas the realistic geometry shows AZ-specific behavior (Figure 7) and a broad range of clearance efficiency depending on the AZ geometry (Figure 7; Table 3) or the distribution of DATs (Figure 9). Below is an excerpt from page 18, describing the new results added to Figure 9G-H.

We investigated the differences among well-mixed, simple spatial (ellipsoid), and complex realistic geometries while keeping the EC volume, surface area and total density of DAT fixed. Results suggest that the approximation of the axonal morphology by a simplified (ellipsoidal) geometry yields results comparable to those predicted by a well-mixed model. Inclusion of realistic geometry, on the other hand, leads to a broad range of results depending on the specific AZ (Figures 7-8) and on the surface distribution of DATs (Figure 9G-H).

5. Further, the authors did not use the model to answer the “why” question: why would dopamine terminals have a heterogeneous DAT distribution when the model reveals this distribution has a lower efficiency of uptake efficiency.

The answer to the “why” question is not within the scope of this study; here the heterogeneous distribution of DAT is deduced directly from image data, and we focused on the effect of the spatial organization of DATs on their DA reuptake efficiency. However, we agree that this is an interesting observation. It is indeed shown and thoroughly discussed in another recent study (Ma et. al. 2017) the fact that the distribution of DATs (wild type and selected mutants) on the surface varies depending on their preferred conformational states. Therein, the local curvature/shape of the membrane is shown to affect the conformational state of the DAT; and DAT molecules either migrate to regions that accommodate their conformation or induce a membrane remodeling upon clustering. For example, outward-facing conformers tend to populate the filopodia, and membrane curvature appears to be a feature driving the non uniform distribution of DATs. We have now expanded the Discussion to discuss these observations (Page 22)

From page 22,

Recent studies indicate that the population of DATs may vary depending on the membrane curvature (Caltagarone et al., 2015) . Notably, selected DAT mutants that have disrupted OF conformations do not accumulate in filopodia, suggesting that access to the OF/OF* state may be a prerequisite for DAT to populate the filopodia (Caltagarone et al., 2015). Further examination showed that binding of cocaine and its fluorescent analog JHC1-64 also alters the plasma membrane distribution of both wild type and mutant DATs. Cocaine binding arrests DAT in the OF state, and cocaine-bound DATs predominantly localize in the filopodia (Ma et al., 2017). Likewise, zinc binding, also known to stabilize the OF state, led to an increase in the level of DAT mutants in the filopodia. These observations suggest that the membrane curvature is a determinant of DAT clustering, with the convex shape of the filopodia favoring the localization of OF DATs. Furthermore, clustering of OF DATs could remodel the membrane to induce an overall outward bending (Ma et al., 2017). The present study suggests that such targeting of axonal protrusions by OF DATs, along with their membrane remodeling capacities, may regulate DA reuptake.

Finally, many acronyms and terms not commonly encountered by a general neuroscience audience are lacking and there are many grammatical errors and typos in the text.

Response: We thank the author for bringing these issues to our attention. We have carefully proofread the manuscript and made necessary corrections.

Reviewer #2

The manuscript describes modeling the spatiotemporal dynamics of the concentration of dopamine released from axon terminals as a function of action potential firing pattern, distance from the release site, the distribution of DAT, and the transport cycle of dopamine through DAT conformations. The data are discussed in the framework of dopamine D1 and D2 receptor activation. The simulations will contribute to the understanding of dopamine levels, especially as more is known about the location of release sites and dopamine receptors.

Response: We thank the reviewer for careful evaluation of our manuscript and for supportive comments and overall positive assessment of the significance of our work. We have addressed each of the comments/suggestions listed below, which improved the clarity and the overall presentation of the revised manuscript.

The following points should be clarified in the text:

1.When initially introduced, [DA]EC, [DA]local, and [DA]syn should be delineated in terms of distance from the release site and time from release event, in the main text.

Response: We clarified the definition of these concentrations as suggested. Below are corresponding excerpts.

page 10

Each blue dot represents the outcome, EC DA concentration in the simulation box, [DA]EC, from one run.

page 13

The curves in Figure 7A display the time evolution of the local concentration, [DA]local, within a spherical shell of 1.5 µm thickness, with respective inner and outer radii of 1 µm and 2.5 µ (i.e. 1 {less than or equal to} r < 2.5 µm) centered at the release site, following a release event from the release site on each of the 6 AZs. Results represent the averages over multiple release events (140 per AZ) observed within the time interval 0 < t < 40 ms.

page 17

We defined [DA]syn as the transient DA level within a sphere of 0.5 µm radius centered at a release site.

2.The “active zones” described in the manuscript are not unequivocal release sites, but assumed to be dopamine release sites. Without the inclusion of more data to this end, this point should be made clear in the text. In addition, the sentence on page 6 “AZs lie within varicosities but are not usually populated with DAT...” should have a reference.

Response: We clarified that each active zone has a unique release site and added a reference (Block et al, 2015, J Neurosci).

page 8

Each AZ has a release site located at its center; and upon a release event, a total of N0 DA molecules is assumed to be released from the release site.

3. It is unclear where postsynaptic D1 and D2 receptors are being assumed to be localized relative to the release site. In the absence of data demonstrating their location (e.g. in brain slices), these assumptions should be described clearly and referenced, in the Results and Discussion.

Response: Thank you for the useful suggestion. We have now clarified that the D1 and D2 receptors are assumed to be uniformly distributed on the plasma membrane of all pre- and postsynaptic cells lining the extracellular region. We have added sentences to both Results (page 15) and Discussion sections to describe this feature and discuss its validity. We also added appropriate references (Dreyer et al, 2010, J Neurosci), (Ford, 2014, Neuroscience) and (Cadet et al, 2013, CNS Neural Disord Drug Targets) to these sentences. Below are corresponding excerpts.

page 15

For the calculation of occupancy, high- and low-affinity receptors were assumed to be uniformly distributed on the membrane of all pre- and postsynaptic cells similar to previous approaches (Dreyer et al., 2010). Note that the activation of low affinity receptors requires high [DA]local values, and such high concentration levels are temporarily attained only in the proximity of the release sites. So, in practice, only those low affinity receptors located sufficiently close to the AZs will be activated. As to high affinity receptors, they are likely to be activated at all locations. To evaluate the fractional occupancy of high affinity DA receptors for each of the 6 AZs, we analyzed 140 x 6 = 840 runs as follows: we divided the simulation box into cubic grids of 1 femtoliter (10-15 liters)

page 23

Previous work showed that the D1 receptors are mostly localized at the post-synaptic membrane (Cadet et al., 2010); and our simulations show that only those low affinity receptors localized near the synapse can be activated. In the case of high-affinity receptors, on the other hand, proximity to release site is not a requirement for being activated; high-affinity receptors located on distal regions may be equally activated.

Minor points:

1. The introduction “low levels of DA cause PD” should be changed to “low levels of DA cause motor impairments associated with PD”.

Response: Changed.

2. It is unclear to what “DA density of the neurons” on page 7 is referring.

Response: We changed “DA density of the neurons” to “content of DA in the striatum” in striatum“.

3. Page 15, “Table 5” should read “Table 3”

Response: Fixed.

4.Discussion of D2 autoreceptor activation/occupancy would broaden the scope and readership of this manuscript.

Response: We extended our discussion on D2 autoreceptors and how it can regulate the DA release and reuptake mechanisms. We have added two new paragraphs along with three new references to page 24, highlighted in blue.
